# Unlocking Cryogenic Self‐Assembly of Lyotropic Liquid Crystals: A Molecular Perspective From Short‐Range to Long‐Range Scales

**DOI:** 10.1002/advs.202512502

**Published:** 2025-09-15

**Authors:** Weiluo Guo, Zhenghua Sun, Runxi Wang, Zhuo Zheng, Yubin Ke, Hua Yang, Lingzhi Xie, Yujun Feng, Hongyao Yin

**Affiliations:** ^1^ Polymer Research Institute State Key Laboratory of Advanced Polymer Materials Sichuan University Chengdu 610065 P. R. China; ^2^ Institute of New Energy and Low‐Carbon Technology Sichuan University Chengdu 610065 P. R. China; ^3^ Spallation Neutron Source Science Center Dongguan 523803 P. R. China; ^4^ Institute of High Energy Physics Chinese Academy of Sciences Beijing 100049 P. R. China

**Keywords:** lamellar liquid crystals, molecular dynamic simulation, phase transition, subzero temperature, supramolecular self‐assembly

## Abstract

Achieving the self‐assembly of lamellar liquid crystals (LLCs) at sub‐zero temperatures and elucidating their structure‐assembly interplay are crucial for understanding cryobiological processes and facilitating cryogenic soft materials; however, this remains a formidable challenge. Herein, six alkyl alkanolamide amphiphiles are designed, and their self‐assembly behavior in 1,2‐propanediol/water cosolvent is investigated from 80 to −20 °C. The hydrocarbon chain length exerts a significant influence on self‐assembly behavior at both short‐range and long‐range scales. Amphiphiles with hydrocarbon chains shorter than C16 (i.e., the number of carbon atom is 16) exhibit limited solubility and cannot form LLCs at low temperatures, while longer chains enhance cryo‐solubility and self‐assembly capabilities, contradicting conventional assumptions. Notably, amphiphiles with chains of C18 or longer require only 0.3 wt.% for LLCs formation. These LLCs exhibit intriguing temperature‐dependent phase transitions, including a liquid‐like lamellar phase, a tilted gel phase, and a distinct phase characterized by tighter alkyl chain packing. The hydrocarbon chain length directly governs the transition temperatures and further influences the long‐range orientational ordering of lamellar sheets. Additionally, the tightly‐packed configuration confers exceptional rheological properties, including ultra‐high viscosity, shear‐thinning behavior, and elasticity. These findings provide important insights for the design and engineering of high‐performance soft materials used in extreme environments.

## Graphical abstract

Cryogenic self‐assembly of lyotropic liquid crystals is achieved using alkyl alkanolamide amphiphiles in a 1,2‐propanediol/water cosolvent system. The hydrocarbon chain length significantly affects the molecular arrangement in bilayers and their long‐range orientational ordering. These insights could advance lipid molecule design for cryo‐stable membranes and guide the engineering of high‐performance soft materials for extreme environments.

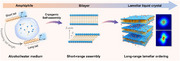

## 1 Introduction

Amphiphilic molecular self‐assembly, a process driven by supramolecular interactions such as hydrophobic effects and hydrogen bonding, enables the spontaneous organization of molecules into ordered nano/micro‐structures.^[^ ^1^ ^–^ ^3^ ^]^ This ubiquitous phenomenon not only underpins essential biological processes, exemplified by the hierarchical architecture of cellular membranes, but also serves as a versatile platform for designing advanced functional materials in nanotechnology, biomedicine, and soft matter systems.^[^ ^4^ ^–^ ^8^ ^]^ Of particular importance are amphiphiles with specific molecular architectures that self‐assemble into lamellar liquid crystals (LLCs), which are distinguished by alternating bilayers and solvent molecular layers.^[^ ^9^, ^10^ ^]^ These periodic nano/micro‐structures have attracted considerable scientific interest due to their functionality in simulating cell membrane,^[^ ^11^ ^]^ encapsulating hydrophobic therapeutics for drug delivery,^[^ ^12^ ^]^ and templating the synthesis of nanostructured materials with precisely controlled morphologies.^[^ ^13^, ^14^ ^]^ However, current research on amphiphilic self‐assembly remains largely restricted to ambient or elevated temperatures, while systematic investigation under subzero conditions—a critical domain for applications in cryopreservation, polar environment engineering, and understanding life's survival at low temperatures—remains markedly underexplored.

Recent advancements in nonconfinement strategies provide promising opportunities to address this limitation.^[^ ^15^, ^16^, ^17^ ^]^ Amphiphile self‐assemblies with narrow spaces can serve as nanoconfinements that confine water in a non‐crystalline state down to subzero temperatures. For example, Mezzenga and coworkers^[^ ^18^ ^]^ demonstrated that cyclopropanated lipids (e.g., monolactobacillin and dicyclopropylmonolinolein) can form liquid crystalline phases capable of confining water in a non‐crystalline state down to −263 °C. This phenomenon arises from geometrically constrained nanochannels within the assemblies, which kinetically inhibit ice nucleation. Their subsequent investigations revealed that phytantriol‐based lipidic mesophases could similarly preserve liquid water down to −120 °C.^[^ ^19^ ^]^ Utilizing this unfreezing subzero water, cryo‐enzymatic reactions and polymerizations were successfully realized.^[^ ^20^ ^]^ Despite these advantages, this method requires exceptionally high concentrations of lipids (> 90 wt.%) to form sufficient nanoconfinements that preserve water in a non‐crystalline state, potentially imposing practical limitations for scalable applications.

The incorporation of freezing‐point inhibitors, such as small molecular alcohols, represents an alternative approach to realizing cryogenic self‐assembly. Alcohols, including methanol, ethanol, ethylene glycol, and glycol, can effectively suppress water crystallization through competitive hydrogen bonds (H‐bonds) formation with water molecules, thereby lowering the freezing point.^[^ ^21^, ^22^ ^]^ However, investigations into the self‐assembly of amphiphilic molecules with alcohol/water cosolvents remain scarce, particularly under sub‐zero conditions. This scarcity primarily arises from two challenges:^[^ ^23^, ^24^ ^]^ the presence of alcohol diminishes the solvophobic interactions essential for amphiphile self‐assembly, thereby compromising assembly efficacy; amphiphilic molecules exhibit significantly reduced solubility at low temperatures. Consequently, despite its pivotal importance for advancing the mechanistic understanding of cryo‐assembly phenomena, cryobiological processes, and the development of cryogenic soft materials, unlocking the self‐assembly of lyotropic liquid crystals (LLCs) at sub‐zero temperatures and elucidating the intricate structure‐assembly interplay remains a formidable challenge.

**Figure 1:**
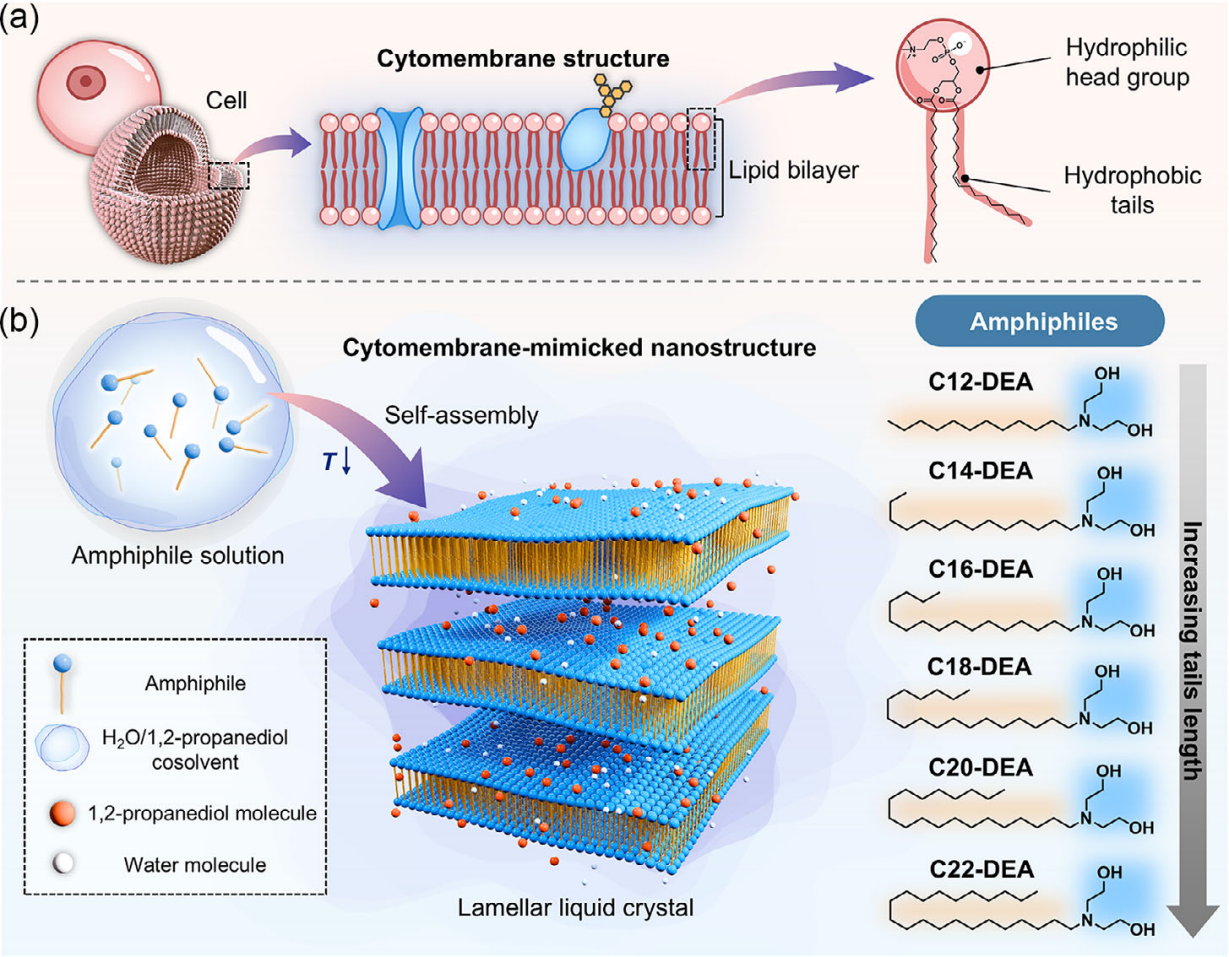
The design strategy of amphiphilic molecules to form lamellar liquid crystals at low temperatures. a) Schematic illustration of phospholipid bilayer in cell membrane. b) Schematic depiction of the microstructure of lamellar liquid crystals and the chemical structure of alkyl diethanolamine amphiphiles used in this work.

Inspired by the phospholipid bilayer in cell membranes (**Figure** **1**a), we designed six alkyl alkanolamide amphiphiles featuring a large hydrophilic head group and varying hydrocarbon chain lengths (Cn‐DEA, Figure 1b) to address this challenge. These amphiphiles are specifically engineered to probe the interplay between molecular structure, nanostructural evolution, and macroscopic rheological behavior. Their self‐assembly behaviors were investigated in 1,2‐propanediol (PG)/water binary systems across a temperature range from 80 to −20 °C. By integrating small‐angle X‐ray scattering (SAXS), cryogenic small‐angle neutron scattering (cryo‐SANS), cryogenic wide‐angle X‐ray scattering (cryo‐WAXS), and molecular dynamics simulations, we elucidated the molecular‐scale mechanisms governing both short‐range and long‐range self‐assembly behaviors over this broad temperature range, and correlated these nanostructural transitions with macroscopic rheological behaviors.

## 2 Results and Discussion

### 2.1 Amphiphiles' Solubility at Low Temperatures

**Figure 2:**
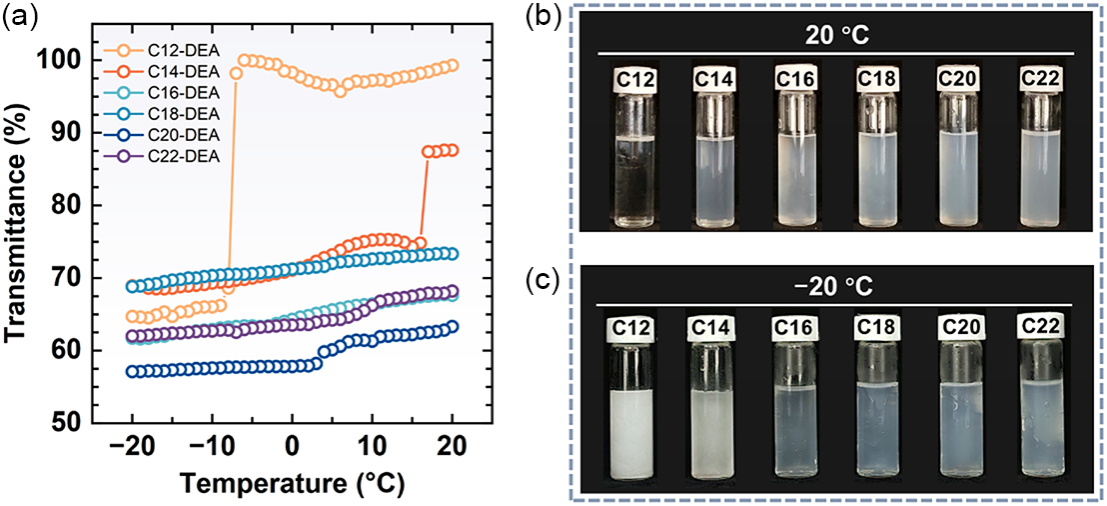
a) Light transmittance at 650 nm for 0.5 wt.% alkyl diethanolamine amphiphiles in 1,2‐propanediol/water (50/50, vol%) as a function of temperature. Visual observations of the mixtures b) at 20 °C and c) at −20 °C after 30 h.

C12‐DEA and C18‐DEA are commercially available, whereas C14‐DEA, C16‐DEA, C20‐DEA, and C22‐DEA were synthesized in the laboratory. The synthesis procedures are provided in the Supporting Information, and the corresponding characterization spectra are presented in Figures S1–S3 (Supporting Information). We initially evaluated the solubility of the six alkyl diethanolamine amphiphiles at temperatures from 20 to −20 °C. PG/water (50/50, vol%) binary solvent was employed as self‐assembly media because of its low freezing point, ≈−37 °C. **Figure** **2**a illustrates the light transmittance at 650 nm of 0.5% alkyl diethanolamine dissolved in the cosolvent as a function of temperature. At 20 °C, the transmittance of all solutions exceeded 60%. Based on visual observations (Figure 2b), no precipitation was evident for any of the amphiphiles, confirming they were fully incorporated into the solution phase. The reduced transparency for systems containing alkyl diethanolamine amphiphiles with longer hydrocarbon chains may indicate the formation of self‐assembled aggregates.^[^ ^25^ ^]^

With the decrease in temperature, the transmittance of C12‐DEA and C14‐DEA significantly decreased from 99% to 66% at −8 °C and from 87% to 75% at 16 °C, respectively. In contrast, solutions containing other amphiphiles with longer hydrocarbon chains exhibited only a minor reduction in transmittance as the temperature was lowered to −20 °C. The temporal variation in the appearance of the solutions at −20 °C is presented in Figure S4 (Supporting Information). The C12‐DEA solution began to form flocculent precipitates after being maintained at −20 °C for 3 h, and it almost completely transformed into undissolved white precipitates after 20 h. Flocculent white precipitates were observed for the C14‐DEA solution after 20 h, becoming more pronounced after 30 h. However, no significant changes were noted in other solutions even after 30 h (Figure 2c). These findings demonstrate that alkyl diethanolamine amphiphiles with shorter hydrocarbon tails (e.g., C12 and C14) exhibit limited solubility at subzero temperatures. This observation contrasts with the conventional understanding that amphiphiles with longer hydrocarbon chains typically display poor solubility.^[^ ^26^ ^]^ This unusual dissolution behavior might be related to the self‐assembly process,^[^ ^27^ ^]^ which will be further elucidated in the subsequent section through molecular dynamics simulation.

### 2.2 Self‐Assembly of LLCs Down to Subzero Temperatures

**Figure 3:**
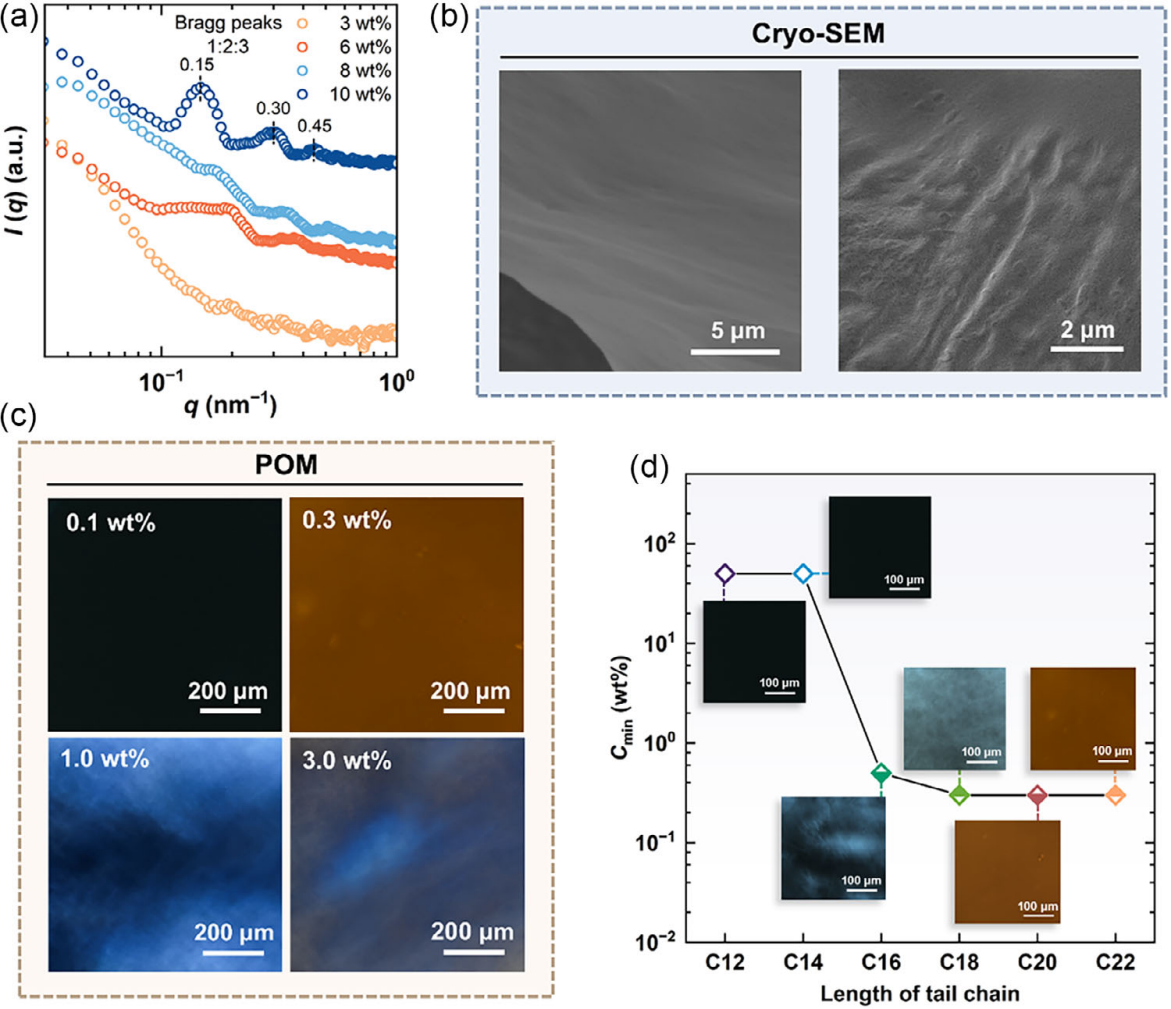
Amphiphile self‐assembled microstructures in 1,2‐propanediol/water (50/50, vol%) binary solvent at 20 °C. a) SAXS profiles for C22‐DEA system at different concentrations. b) Cryo‐SEM images for 5.0 wt.% C22‐DEA system. c) POM images for C22‐DEA system at different concentrations. d) Minimum amphiphile concentration required for forming LLCs. Hollow icons indicate LLCs do not form at this concentration.

C22‐DEA was chosen as a representative amphiphile to investigate the self‐assembly behavior at 20 °C. **Figure** **3**a presents the SAXS profiles of C22‐DEA across a concentration range from 3.0 to 10.0 wt.%. Bragg peaks appeared when the C22‐DEA concentration reached 6.0 wt.%, and both the intensity and sharpness of these peaks progressively increased as the concentration was raised from 6.0 to 10.0 wt.%. For the system containing 10.0 wt.% C22‐DEA, distinct Bragg peaks with relative positions of 1:2:3 were observed, indicating the formation of LLCs.^[^ ^28^ ^]^ It is concluded that the structural regularity of the LLCs improves with increasing C22‐DEA concentration. Analysis of the Bragg peaks revealed that the interlamellar spacing (*d*_sp_) was ≈42 nm in this case.

Cryogenic scanning electron microscopy (cryo‐SEM) and polarizing optical microscopy (POM) were then employed to directly examine the assembled morphology. Cryo‐SEM images revealed tightly stacked lamellar structures in the system containing 5.0 wt.% C22‐DEA (Figure 3b). Figure 3c presents the POM images of the systems with varying C22‐DEA concentrations. No discernible texture was observed for the sample with only 0.1 wt.% C22‐DEA, whereas oily streak textures emerged in samples at the concentrations of 0.3 wt.% or higher. Given the high sensitivity and maneuverability of POM for observing LLCs, it was subsequently employed to determine the minimum concentration required for these amphiphiles to form LLCs. As shown in Figure 3d, significant variations in self‐assembly capabilities were observed among them. Specifically, amphiphiles with shorter hydrophobic chains exhibited poorer self‐assembly abilities in forming LLCs. Notably, C12‐DEA and C14‐DEA failed to form LLCs even at a concentration of 50.0 wt.%. In contrast, the minimum concentration required for C16‐DEA was only 0.5 wt.%, and it further decreased to 0.3 wt.% for C18‐DEA, C20‐DEA, and C22‐DEA.

**Figure 4:**
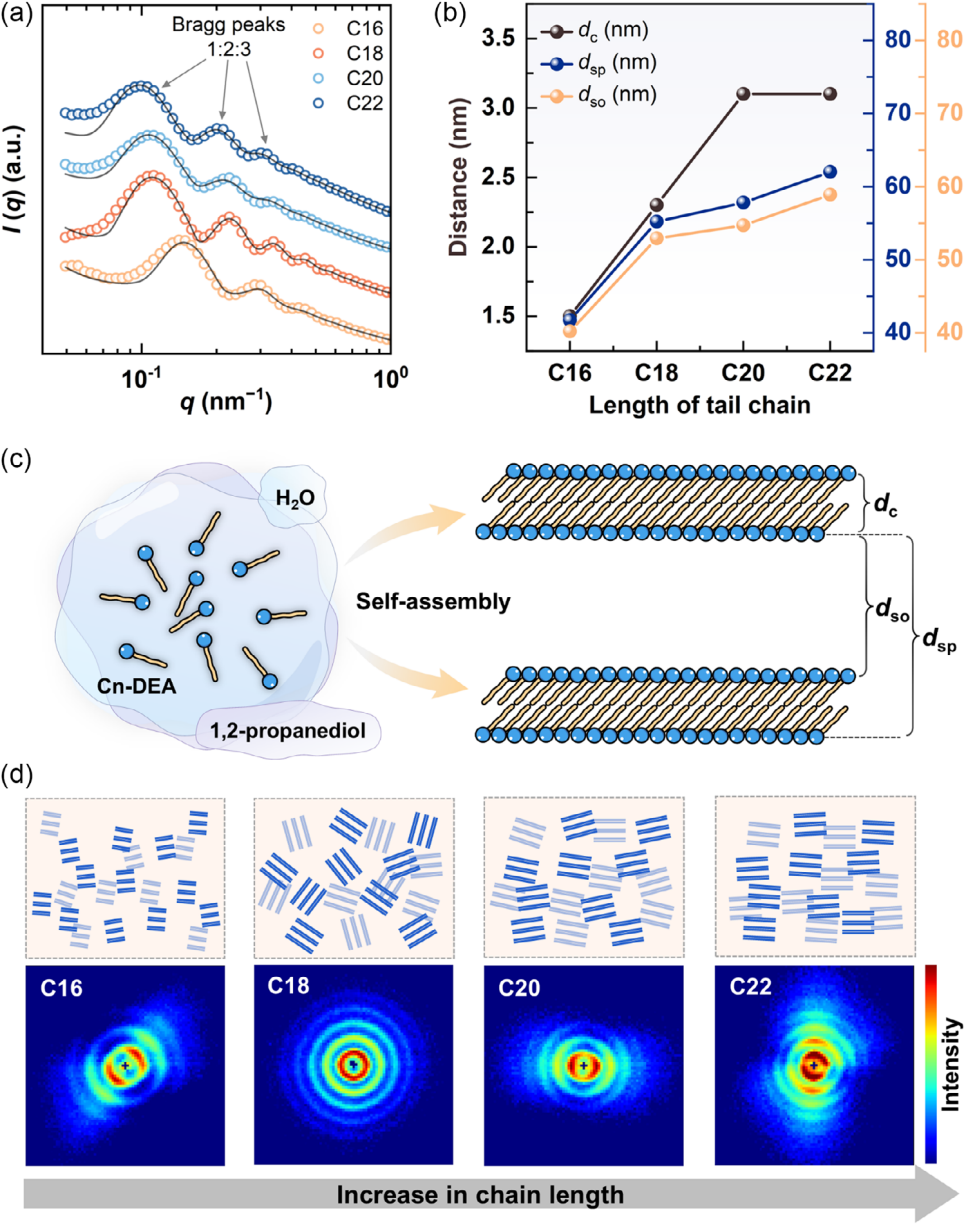
a) 1D SANS profiles for 5.0 wt.% alkyl diethanolamine amphiphiles in 1,2‐propanediol/deuterium oxide (50/50, vol%) at 20 °C. b) Structural parameters of interlamellar spacing (*d*_sp_), bilayer thickness (*d*_c_), and solvent layer thickness (*d*_so_) for LLCs formed by different amphiphiles. c) Schematic diagram for the self‐assembly process of LLCs with tilted gel phase (L_β'_). d) 2D SANS patterns and the corresponding schematic illustration of the long‐range arrangement of lamellar sheets.

SANS was further employed to elucidate the distinct self‐assembly behavior of the four amphiphiles at a concentration of 5.0 wt.%. Bragg peaks with relative position of 1:2:3 were clearly observed in the 1D SANS profiles (**Figure** **4**a), which strongly indicate the formation of LLCs. This finding is consistent with the results obtained from SAXS analysis. As the hydrocarbon chain length increases, the Bragg peaks progressively shift toward lower *q* values, reflecting an increase in interlamellar spacing (*d*_sp_). The entire SANS profile was analyzed using the lamellar stack Caille model within the SasView software to achieve an optimal goodness‐of‐fit. The structural parameters derived from the fits are summarized in Table S1 (Supporting Information) and depicted in Figure 4b. The results revealed that the bilayer thickness (*d*_c_) was 1.5 nm for C16‐DEA, increasing to 2.3 nm for C18‐DEA, and further rising to 3.1 nm for both C20‐DEA and C22‐DEA. It exhibits a linear relationship with the hydrophobic chain length for C16‐DEA, C18‐DEA, and C20‐DEA; however, this trend ceases for C22‐DEA. This increase can be readily attributed to the elongation of the hydrocarbon tail, which directly contributes to the increased bilayer thickness. Nevertheless, when the hydrocarbon tail becomes sufficiently long, it tends to adopt a more flexible conformation,^[^ ^29^ ^–^ ^31^ ^]^ thereby preventing a significant increase in bilayer thickness (Figure S7, Supporting Information). It is worth noting that the measured bilayer thickness values are significantly smaller than the geometric length scale of the corresponding pair of hydrocarbon tails. For example, the tail length of C22‐DEA is 1.8 nm at 20 °C (Figure S8, Supporting Information), while the thickness of the corresponding bilayer is only 3.1 nm. This discrepancy may suggest that the LLCs exist in a tilted gel phase (L_β'_), where the molecular pairs are packed in a tilted configuration within the lamellar sheets (Figure 4c).

In addition, the interlamellar spacing *d*_sp_ exhibited a significant dependence on variations in hydrocarbon tail length. It was 41.7 nm for C16‐DEA, increased dramatically to 55.2 nm for C18‐DEA, and then rose gradually to 57.8 nm for C20‐DEA and 62.0 nm for C22‐DEA. These values are comparable to those obtained from SAXS analysis, suggesting that the model is accurate and reliable. The *d*_sp_ can be viewed as the sum of *d*_c_ and solvent layer thickness (*d*_so_), as illustrated in Figure 4c. Consequently, the solvent thickness can be quantitatively determined using the formula *d*_so_ = *d*_sp_ − *d*_c_. The variation of *d*_so_ with changes in hydrophobic tail length is presented in Figure 4b. Compared to the *d*_c_, the *d*_so_ is approximately one order of magnitude larger, thereby playing a dominant role in determining the *d*_sp_. Furthermore, the *d*_so_ increases systematically with the elongation of the hydrophobic alkyl chain, exhibiting a trend consistent with the variation in interlamellar spacing. This phenomenon can be attributed to the following factors: At an identical mass concentration, alkyl diethanolamine amphiphiles with shorter hydrocarbon chains (e.g., C16‐DEA) contain a greater number of molecules due to their lower molecular weight, resulting in LLCs with a higher density of bilayers and consequently a smaller *d*_so_. Moreover, the increase in hydrocarbon chain length enhances the self‐assembly capability, leading to a more compact molecular packing within the bilayer, which further contributes to the observed increase in *d*_so_.

In comparison with most previously reported LLCs,^[^ ^11^, ^28^, ^32^ ^–^ ^35^ ^]^ both the interlamellar spacing and solvent thickness are significantly larger, by approximately one order of magnitude. Meanwhile, the concentration required to form LLCs is remarkably lower, being approximately two orders of magnitude smaller. These results highlight the exceptional capability of these alkyl alcoholamine amphiphiles to form LLCs.

The 2D SANS patterns of C16‐DEA, C18‐DEA, C20‐DEA, and C22‐DEA were analyzed and are presented in Figure 4d. Notably, the patterns obtained from C16‐DEA, C20‐DEA, and C20‐DEA exhibited strong anisotropy, indicative of long‐range orientationally ordered lamellar structures. The scattering pattern of C16‐DEA revealed three observable reflection orders, while those of C20‐DEA and C22‐DEA displayed four diffraction peaks, demonstrating a higher degree of structural order in their LLCs. In contrast, the 2D SANS pattern of C18‐DEA was isotropic with four orders of reflection visible, suggesting randomly packed lamellar sheets, which was distinctly different from the other three systems. These findings underscore the critical role of hydrocarbon chain length in both the formation of LLCs and the long‐range orientational arrangement of lamellar sheets.

**Figure 5:**
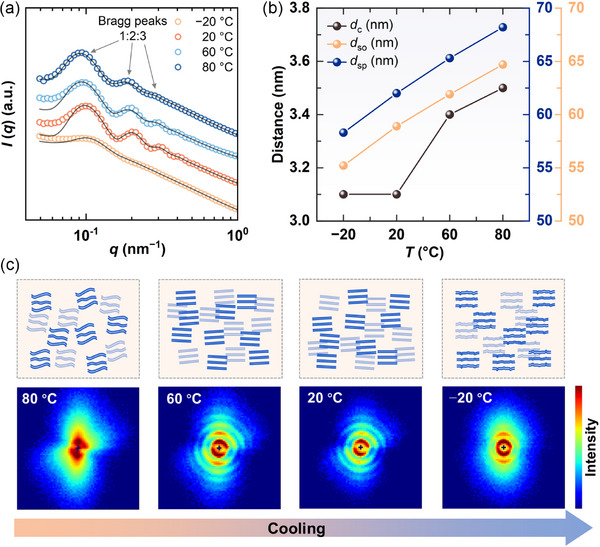
SANS characterization of 5.0 wt.% C22‐DEA in 1,2‐propanediol/deuterium oxide (50/50, vol%) at various temperatures. a) 1D SANS profiles. b) Structural parameters of interlamellar spacing (*d*_sp_), bilayer thickness (*d*_c_), and solvent thickness (*d*_so_) as a function of temperature. c) 2D SANS images at various temperatures, along with their corresponding schematic representation of the long‐range arrangement of lamellar sheets.

We subsequently investigated the evolution of self‐assembled microstructures as the temperature was decreased from 80 to −20 °C. **Figure** **5**a illustrates the 1D SANS profiles of 5.0 wt.% C22‐DEA at various temperatures. The scattering patterns were fitted using the same lamellar stack Caille model in SasView software, achieving a satisfactory goodness‐of‐fit, confirming the presence of LLCs across this entire temperature range. The resulting structural parameters obtained from the fits are depicted in Figure 5b and summarized in Table S2 (Supporting Information). The bilayer thickness was 3.5 nm at the higher temperature of 80 °C, decreased to 3.4 nm at 60 °C, further reduced to 3.1 nm at 20 °C, and remained constant at −20 °C. Additionally, the *d*_so_ also diminished from 64.7 nm at 80 °C to 55.2 nm at −20 °C, showing an interesting linear relationship. These observations can be attributed to the more compact packing of C22‐DEA molecules as the temperature decreased. It is evident that reducing the temperature promotes the self‐assembly of alkyl alcoholamine amphiphiles, resulting in enhanced molecular packing density.

The influence of temperature on the orientation of lamellar sheets is distinctly reflected in the 2D SANS patterns (Figure 5c). At the higher temperature of 80 °C, the sample exhibited an irregular 2D pattern with indistinct diffraction peaks, indicating that the lamellar sheets were coarsely orientated in one direction while the molecules within the sheets may lack ordered packing. This phenomenon can be attributed to the enhanced thermal motion of C22‐DEA molecules and the reduced number of H‐bonds between C22‐DEA and solvent molecules at elevated temperatures, which are insufficient to effectively confine the C22‐DEA molecules within the solvo‐phobic region. Upon reducing the temperature to 60 and 20 °C, the patterns showed strong anisotropy, and the diffraction peaks became clearer, suggesting the formation of regular and ordered packing of molecules in bilayers as well as the long‐range orientational ordering of lamellar sheets. At −20 °C, the 2D pattern exhibited significant anisotropy, with high intensity along the vertical direction, which implies that the lamellar sheets were strongly aligned in a single direction. However, the diffraction peaks became indistinct again, reflecting a reduction in the packing order of C22‐DEA molecules within bilayers. It is interesting that as the temperature reduced from 80 to −20 °C, the molecular packing order initially increased and subsequently decreased in the bilayer, while the overall packing of lamellar sheets consistently improved.

**Figure 6:**
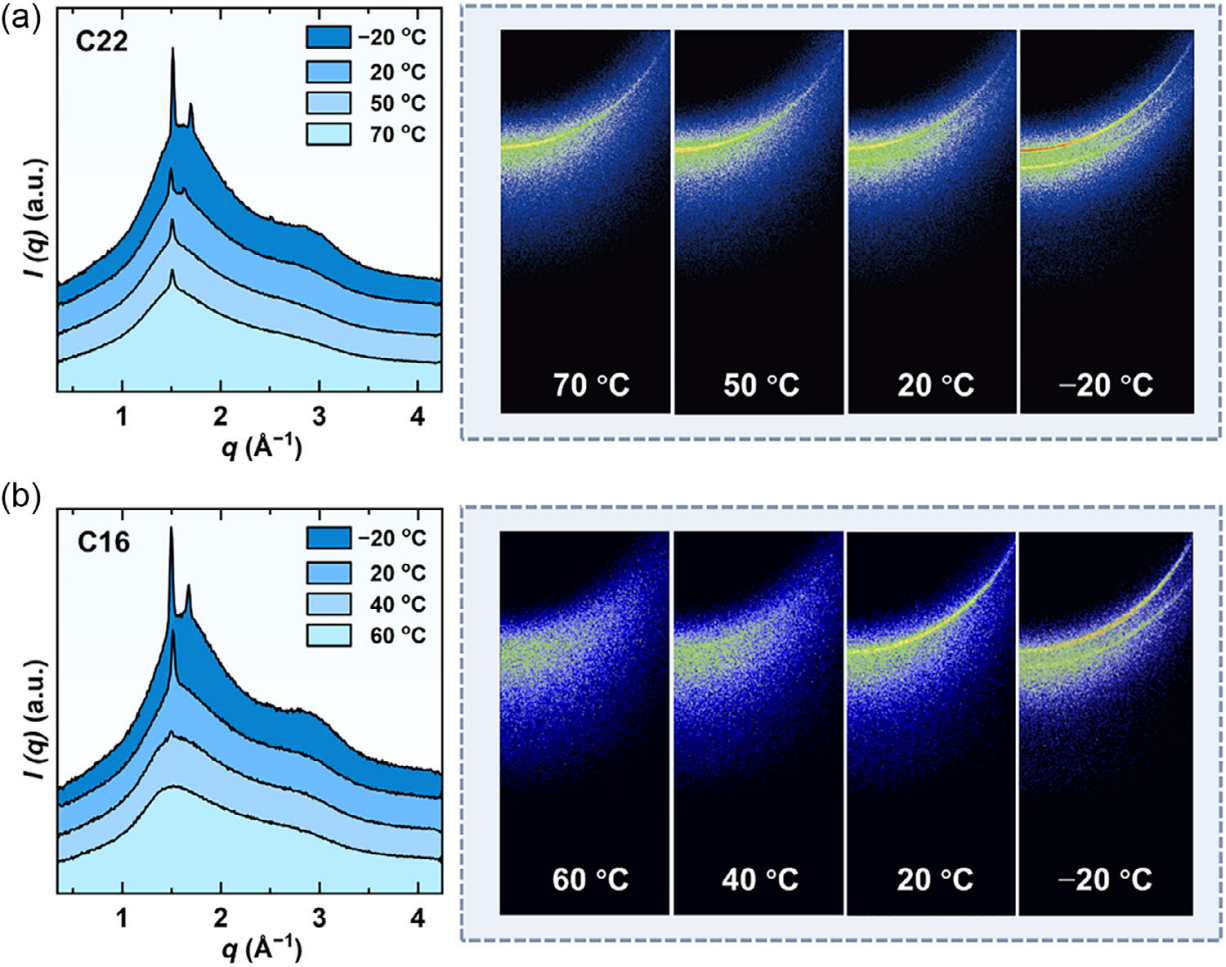
1D WAXS profiles and 2D WAXS images of 5.0 wt.% a) C22‐DEA and b) C16‐DEA in 1,2‐propanediol/Water (50/50, vol%) at various temperature.

To gain an insight into the changes in the precise microstructure of the assemblies as the temperature decreases, WAXS measurements were conducted. **Figure** **6**a illustrates the 1D WAXS profile and 2D WAXS images of a 5.0 wt.% C22‐DEA sample across temperatures ranging from 70 to −20 °C. At 70 °C, a sharp crystalline peak was observed at *q* = 1.51 Å^−1^ in the 1D profile, accompanied by a Deby diffraction arc in the 2D image. This indicates that the LLCs were in the gel phase, where the alkyl chains forming the bilayer were packed in an ordered state. This observation is consistent with the SANS results that the LLCs were in the titled gel L_β'_ phase. As the temperature decreased to 20 °C, a second peak emerged at *q* = 1.63 Å^−1^ in the 1D profile, along with a new outer diffraction arc in the 2D image. Upon further cooling to −20 °C, the intensity of this peak increased significantly, and the diffraction arc became more pronounced. The first peak at *q* = 1.51 Å^−1^ corresponds to a chain‐to‐chain distance of 0.42 nm within the bilayer, while the second peak at *q* = 1.63 Å^−1^ represents an inter‐acyl chain distance of 0.38 nm, implying a distinct configuration with tighter alkyl chain packing emerged. It is worth noting that these two configurations coexisted simultaneously, contrasting with most other systems that generally possess only one alkyl chain configuration. This coexistence explains the reduced molecular packing order within the bilayers, as evidenced by 2D SANS pattern.

Similarly, the 5.0 wt.% C16‐DEA sample was also characterized using the WAXS technique (Figure 6b). In contrast to the results for C22‐DEA, a broad peak was observed in the 1D scattering curve at 60 °C, indicative of the lamellar structures formed by C16‐DEA exhibiting a lamellar liquid‐crystalline (L_α_) phase characterized by disordered alkyl chain packing. When the temperature decreased to 40 °C, a weak peak appeared at the same position of *q* = 1.51 Å^−1^ and became more pronounced as the temperature further decreases to 20 °C, indicating the occurrence of the L_α_−L_β'_ phase transition upon cooling. Interestingly, a new peak at *q* = 1.63 Å^−1^ in the 1D profile and a new outer diffraction arc in the 2D image were also observed when the temperature was further decreased to −20 °C, suggesting the formation of a similar distinct configuration.

In comparison with C22‐DEA system, the C16‐DEA system exhibited identical chain‐to‐chain lengths as temperature decreased. However, the temperatures for phase transition are different. Amphiphiles with longer hydrocarbon chains are more likely to form ordered packing within a bilayer. For instance, C22‐DEA forms LLCs in the L_β'_ phase even at a high temperature of 70 °C, while C16‐DEA requires a temperature reduction below 40 °C to form the L_β'_ phase. It can be concluded that the phase state in LLCs can be regulated through the synergistic effects of hydrocarbon tail length of amphiphiles and variations in temperature.

### 2.3 Molecular Dynamic Simulation

The above results clearly demonstrate that the hydrocarbon chain length of amphiphiles plays a crucial role in determining the solubility at subzero temperatures and governing both short‐/long‐range self‐assembly behaviors. Molecular dynamics simulation serves as a powerful tool for elucidating the underlying mechanism.^[^ ^36^ ^]^ C12‐DEA, C18‐DEA, and C22‐DEA were selected as representative amphiphiles for this investigation. The schematic diagram of the simulation system is presented in Figures S5 and S6 (Supporting Information).

**Figure 7:**
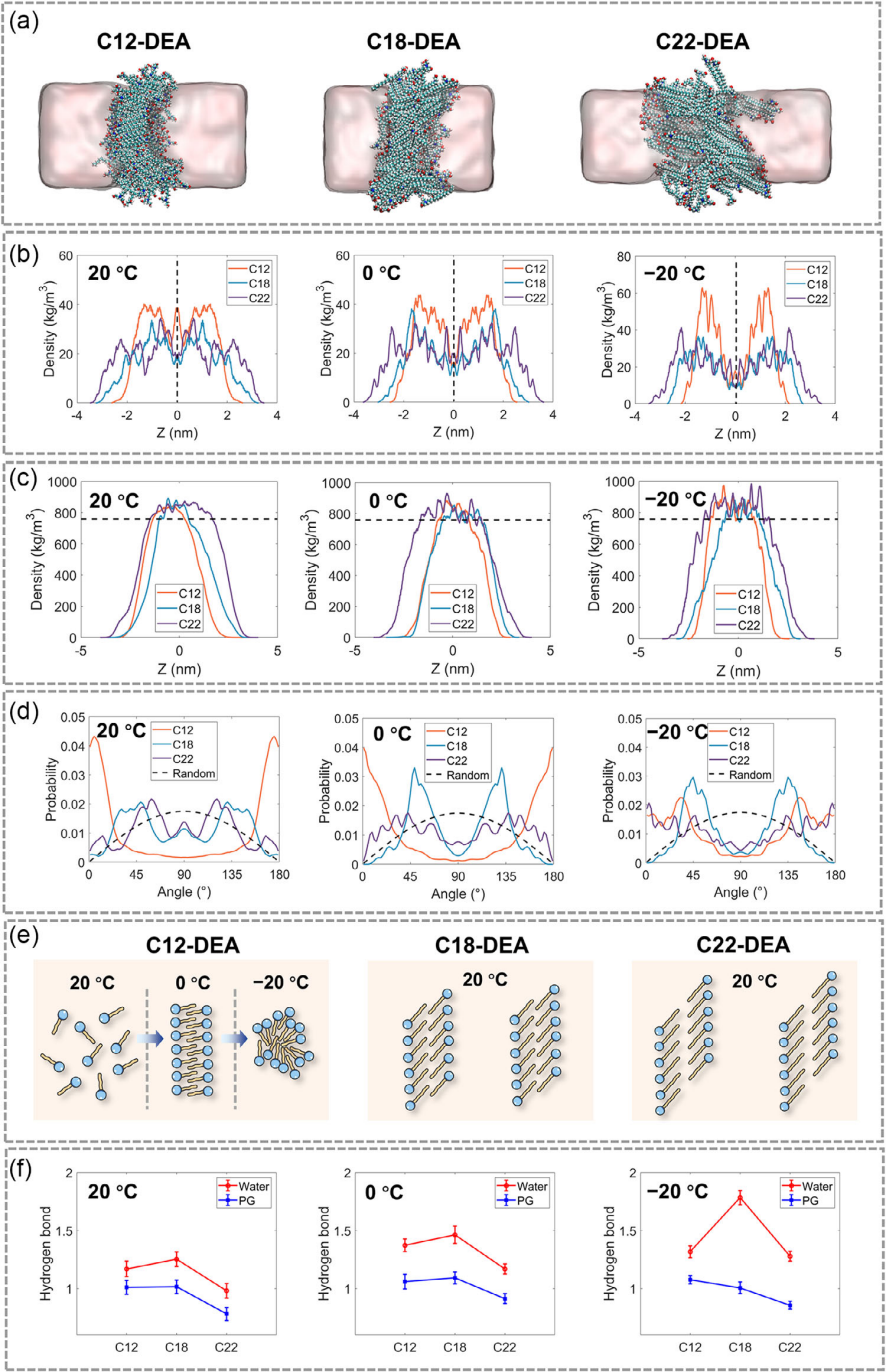
a) Simulation snapshots of C12‐DEA, C18‐DEA, and C22‐DEA in 1,2‐propanediol/water (50/50, vol%) at 20 °C. b) Density distributions of nitrogen atoms, c) density distributions of amphiphile molecules, and d) angles between the amphiphile molecules and the Z‐axis at various temperatures. e) Schematic diagrams of bilayers for C12‐DEA, C18‐DEA, and C22‐DEA. f) Number of H‐bonds between amphiphiles and solvent molecules at different temperatures.

From the simulation snapshots presented in Figure 7a, it was observed that the morphology of C12‐DEA aggregates remained ambiguous, whereas C18‐DEA and C22‐DEA formed well‐defined bilayers. Figure 7b exhibits the density distribution of *N* atoms located at the hydrophilic head group of amphiphilic molecules. The *N* atom density in C12‐DEA was significantly distributed in the center, indicating that C12‐DEA molecules failed to form a regular bilayer. This observation aligns with experimental results, where LLCs were not detected in the C12‐DEA system. In contrast, C18‐DEA and C22‐DEA molecules exhibited a bimodal distribution on opposite sides of the center point on the Z‐axis, confirming the formation of bilayer structures (Figure 7a). These results are consistent with experimental observations. Simulation results revealed that at 20 °C, the hydrocarbon chain lengths of C18‐DEA and C22‐DEA were ≈1.9 and 1.8 nm (Figure S8, Supporting Information), respectively. Analysis of the molecular density profiles (Figure 7c) and the corresponding *N* atom density profiles for C18‐DEA and C22‐DEA indicates that the thicknesses of the bilayers they formed were ≈1.5 nm and 3.2 nm (almost exactly with the experiment value), respectively. In conjunction with the simulation snapshots, it is suggested that these molecules self‐assemble into LLCs with tilted gel L_β'_ phase, which is consistent with experimental results.

The angles between the three amphiphilic molecules and the *z*‐axis direction are illustrated in **Figure** **7**d and were statistically analyzed, which can more precisely reflect their arrangement in the bilayers. At 20 °C, the arithmetic weighted average angles of C12‐DEA, C18‐DEA, and C22‐DEA molecules were 19°, 48°, and 52°, respectively. That is, C18‐DEA and C22‐DEA molecular pairs were arranged more obliquely along the *z*‐axis direction compared to C12‐DEA molecules (Figure 7e). Based on the hydrocarbon tail length and arranged angles, the ideal bilayer thickness (precisely and closely arranged in opposition) can be calculated, which was 2.4 nm for C18‐DEA system and 2.0 nm for C22‐DEA system. However, the thickness obtained from SANS measurements was 2.3 nm for C18‐DEA system and 3.2 nm for C22‐DEA system. This result may suggest that the hydrocarbon chains were slightly interdigitated for C18‐DEA molecules in bilayers, while no interdigitation of hydrocarbon chains occurred for C22‐DEA molecules (Figure 7e).

Upon decreasing the temperature to 0 °C, the density profile of *N* atoms in C12‐DEA exhibited a transformation into a symmetric distribution on both sides of the *z*‐axis center, signifying the onset of bilayer formation. Calculations from the hydrophobic tail length and arrangement angle between C12‐DEA molecules and Z‐axis show that the ideal bilayer thickness was 2.4 nm in this case, which is much larger than the value of 1.6 nm obtained from simulation result. Thus, it is inferred that the hydrocarbon chains in C12‐DEA were arranged significantly interdigitated at 0 °C. When the temperature decreased to −20 °C, the *N* atom density profile of C12‐DEA exhibited narrower and more symmetric peaks compared to those of C18‐DEA and C22‐DEA (Figure 7b), reflecting a more regular and tightly packed molecular arrangement and the significantly reduced molecular thermal motion.

The statistical data of H‐bonds indicates that, as the temperature decreased from 20 to −20 °C, the average number of H‐bonds formed between a C12‐DEA molecule and the solvent molecules increased from 2.2 to 2.4 (Figure 7f). Meanwhile, its hydrophobic tail length decreased from 1.7 to 1.1 nm (Figure S8, Supporting Information). The increased number of H‐bonds more effectively confined the C12‐DEA molecules within the solvo‐phobic regions, while the shortening of the hydrophobic tail weakened the intermolecular interactions. This dual effect likely accounts for the insolubility of C12‐DEA and C14‐DEA at low temperatures (Figure 7e).

On the contrary, C18‐DEA and C22‐DEA, which feature longer hydrocarbon tails, exhibited more significant molecular thermal motion. At −20 °C, the length of their hydrophobic tails was ≈1.3 and 1.5 nm, respectively, much longer than that of C12‐DEA. Analysis of their molecular density and *N* atom density profiles revealed that even at −20 °C, C18‐DEA and C22‐DEA molecules retained considerable mobility within a certain range. Consequently, they remained well‐dissolved at sub‐zero temperatures.

It is important to note that at 20 °C, C18‐DEA exhibited both a longer hydrophobic tail length and a greater number of H‐bonds compared to C12‐DEA and C22‐DEA. This may imply that its molecular thermal motion and self‐assembly behavior are in a critical state: capable of forming lamellar sheets but lacking sufficient energy to further establish long‐range orientationally ordering. This might be accounted for by its isotropic characteristics reflected in the 2D SANS pattern at 20 °C. In contrast, C16‐DEA may form relatively smaller lamellar sheets with long‐range orientational order, while C20‐DEA and C22‐DEA can form larger lamellar sheets exhibiting long‐range orientational order (Figure 4d).

Both experimental and molecular dynamics simulation results confirm the crucial role of molecular architecture in determining the cryogenic self‐assembly behavior. Amphiphiles bearing short hydrophobic hydrocarbon chains exhibit not only limited self‐assembly capability but also poor solubility at low temperatures. Increasing the hydrophobic chain length endows these amphiphiles with the ability to spontaneously form LLCs across a broad thermal range, from elevated to subzero temperatures. Importantly, the hydrophobic chain length not only determines the LLCs phase state and the associated phase transition temperature but also influences the long‐range ordering of lamellar sheets. The observed structure‐property relationships in these amphiphiles suggest that similar effects may occur in naturally occurring lipids, such as phospholipids, which are the predominant constituents of cellular membranes and consist of a large hydrophilic headgroup paired with extended hydrophobic hydrocarbon tails.^[^ ^37^, ^38^ ^]^

### 2.4 Rheological Characteristics

**Figure 8:**
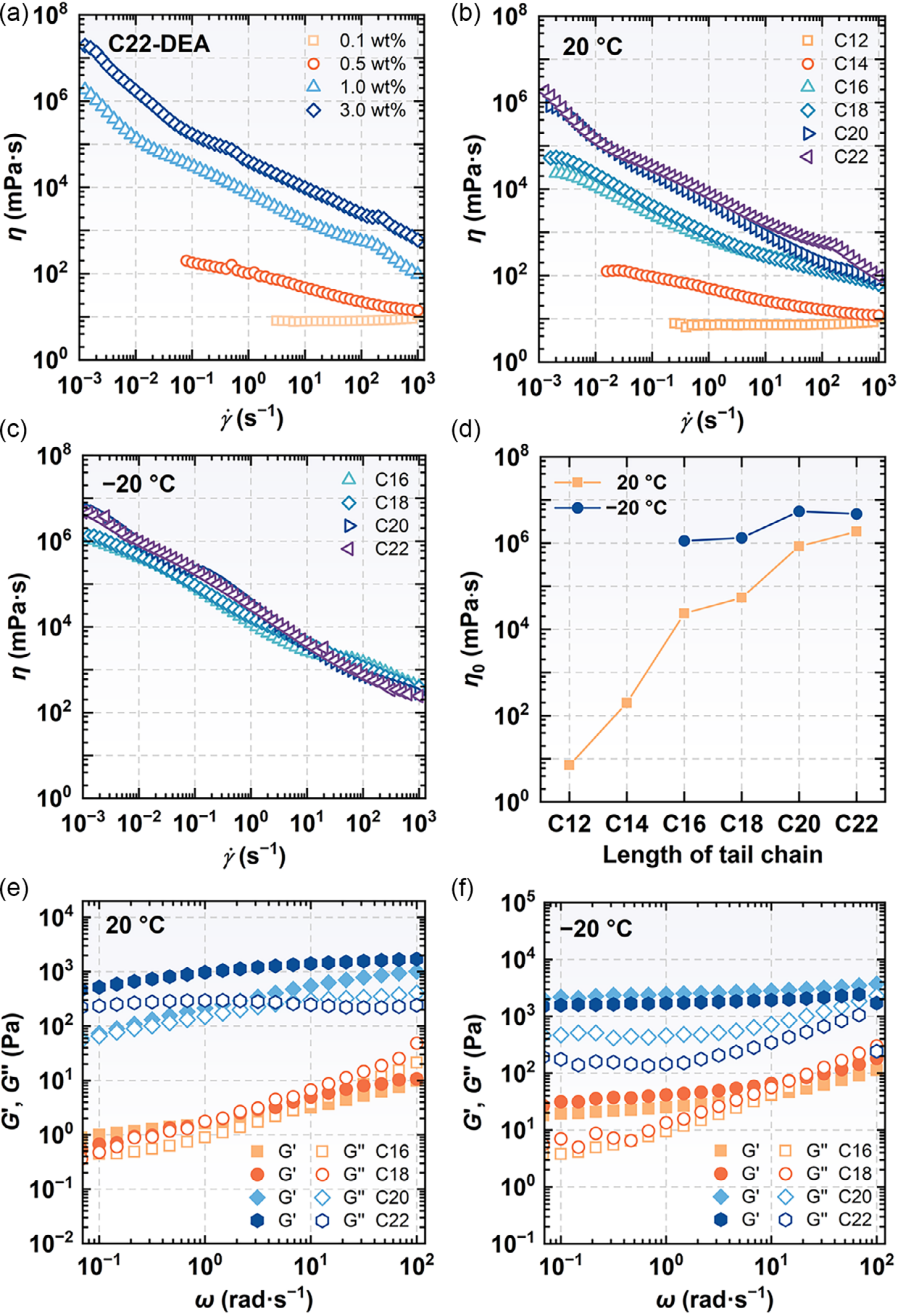
a) Flow curves of C22‐DEA in 1,2‐propanediol/water (50/50, vol%) at different concentrations at 20 °C. Flow curves of 1.0 wt.% amphiphiles in 1,2‐propanediol/water (50/50, vol%) at b) 20 °C and c) −20 °C. d) The zero‐shear viscosity as a function of alkyl chain length. Dynamic rheology of 1.0 wt.% amphiphiles in 1,2‐propanediol/water (50/50, vol%) at e) 20 °C and f) −20 °C.

**Figure** **8**a illustrates the flow curves of C22‐DEA system at 20 °C. At the concentration of 0.1 wt%, the system exhibited a constant low viscosity of 8 mPa·s irrespective of shear rate variations. However, when the concentration increased to 0.5 wt% or higher, the viscosity rose significantly, demonstrating pronounced shear thinning behavior characteristic of non‐Newtonian fluid. This phenomenon is attributed to the formation of LLCs when C22‐DEA concentration exceeds 0.3 wt.%. The pronounced shear thinning behavior can be explained by the shear‐induced alignment of lamellar sheets along the flow direction.^[^ ^32^, ^39^ ^]^ Figure S9 (Supporting Information) depicts the zero‐shear viscosity (*η*_0_) of the fluids as a function of C22‐DEA concentration. Notably, the *η*_0_ became extremely high at concentrations of 1.0 wt% or greater, reaching 1.8 × 10^6^ mPa·s at 1.0 wt.%, which is significantly higher than that of conventional amphiphile self‐assemblies.

Figure 8b presents a comparison of the flow curves for systems comprising 1.0 wt% amphiphiles, where distinct flow behaviors were observed. The C12‐DEA system exhibited a low constant viscosity without shear thinning, whereas the C14‐DEA system demonstrated a *η*_0_ value of 125 mPa·s, accompanied by the onset of shear thinning. Conversely, the C16‐DEA and C18‐DEA systems showed a large increase in the viscosity, with *η*_0_ reaching 2.3 × 10^4^ mPa·s and 5.2 × 10^4^ mPa·s, respectively. Moreover, the C20‐DEA and C22‐DEA systems exhibited a further dramatic rise in viscosity, with *η*_0_ values of 8.5 × 10^5^ mPa·s and 1.8 × 10^6^ mPa·s, respectively. This significant rise in viscosity can be attributed to the differences in alkyl chain configurations within LLCs. At 20 °C, C16‐DEA forms LLCs in a tilted gel L_β'_ phase with a chain‐to‐chain distance of 0.42 nm within the bilayer, whereas the C22‐DEA forms LLCs adopt a configuration characterized by tighter molecular packing, with two chain‐to‐chain distances measured at 0.42 and 0.38 nm, respectively. This structural configuration also influences dynamic rheological properties. As depicted in Figure 8e, a crossover between the storage modulus (*G*′) and loss modulus (*G*″) was observed for both the C16‐DEA and C18‐DEA systems, indicating viscoelastic fluid behavior. In contrast, for C20‐DEA and C22‐DEA systems, The *G*′ remained consistently higher than the *G*″ throughout the accessible frequency range, suggesting gel‐like characteristics.

When the temperature was decreased to −20 °C, both the viscosity and viscoelasticity of the C16‐DEA, C18‐DEA, C20‐DEA, and C22‐DEA systems enhanced significantly (Figure 8c,d,f). The viscosities of all the systems exceeded 1 × 10^6^ mPa·s, accompanied by improved shear‐thinning behavior. The *G*′ of C20‐DEA and C22‐DEA systems increased to over 1 × 10^3^ Pa and greater than *G*″, and the frequency crossover for C16‐DEA and C18‐DEA shifted from 6.8 and 1.0 rad·s^−1^ to 10 and 14.7 rad·s^−1^, respectively. These results can be attributed to the enhanced self‐assembled microstructures at subzero temperatures.

The increase in hydrophobic chain length facilitates the formation of LLCs with a unique configuration with tighter alkyl chain packing. This, in turn, confers the system with superior rheological properties, including extremely high viscosity, pronounced shear thinning behavior, and substantial elastic modulus over a broad temperature range. These findings provide critical insights into the design of various cryogenic soft materials, which hold promise for extensive applications in extreme environments.

## 3 Conclusion

We have designed six alkyl alkanolamide amphiphiles and investigated their solubility, self‐assembly behavior, and macroscopic rheological properties from elevated temperatures to subzero conditions. Amphiphiles with hydrocarbon chains shorter than C16 cannot form LLCs and exhibit poor low‐temperature solubility. Conversely, those bearing longer hydrocarbon chains demonstrated excellent solubility even at −20 °C, and crucially, the extended tail length promotes LLCs formation at an extremely low concentration of 0.3 wt.%. Upon cooling to subzero temperatures, these LLCs phase undergo phase progressive transformations, yielding L_α_, L_β'_, and notably, a distinct phase configuration characterized by tighter alkyl chain packing. Concurrently, the long‐range orientational ordering of the lamellar sheets was significantly improved as the temperature reduced. The hydrocarbon chain length is identified as a pivotal structural parameter, critically governing both the phase transition temperatures and the degree of long‐range lamellar ordering. Furthermore, the formation of the tightly packed phase imparts the system with exceptional rheological properties, including ultrahigh viscosity, pronounced shear‐thinning behavior, and enhanced elastic modulus. These findings provide fundamental insights into the intricate relationship between molecular architecture and low‐temperature self‐assembly mechanisms. We believe this knowledge offers valuable guidance for the rational design and development of high‐performance soft materials engineered for demanding applications in subzero environments.

## Conflict of Interest

The authors declare no conflict of interest.

## Supporting information



Supporting Information

Supporting Information

## Data Availability

The data that support the findings of this study are available from the corresponding author upon reasonable request.
