# Supplementary material for: Unlocking Cryogenic Self‐Assembly of Lyotropic Liquid Crystals: A Molecular Perspective From Short‐Range to Long‐Range Scales
Source: Adv Sci (Weinh). 2025 Sep 15;12(45):e12502. doi: 10.1002/advs.202512502 (PMC12677639; doi:10.1002/advs.202512502)
Supplement: Supplementary file 1 — Supporting Information [file ADVS-12-e12502-s001.docx]

Supporting Information
©Wiley-VCH 2021
69451 Weinheim, Germany

Unlocking Cryogenic Self-Assembly of Lyotropic Liquid Crystals: A Molecular Perspective from Short-Range to Long-Range Scales

Weiluo Guo,^a,1^ Zhenghua Sun,^a,1^ Runxi Wang,^b,^* Zhuo Zheng,^a^ Yubin Ke,^c,d^ Hua Yang,^c,d^ Lingzhi Xie,^b^ Yujun Feng,^a^ Hongyao Yin^a,^*

**Abstract:** Achieving the self‐assembly of lamellar liquid crystals (LLCs) at sub‐zero temperatures and elucidating their structure‐assembly interplay are crucial for understanding cryobiological processes and facilitating cryogenic soft materials; however, this remains a formidable challenge. Herein, six alkyl alkanolamide amphiphiles are designed, and their self‐assembly behavior in 1,2‐propanediol/water cosolvent is investigated from 80 to −20 °C. The hydrocarbon chain length exerts a significant influence on self‐assembly behavior at both short‐range and long‐range scales. Amphiphiles with hydrocarbon chains shorter than C16 (i.e., the number of carbon atom is 16) exhibit limited solubility and cannot form LLCs at low temperatures, while longer chains enhance cryo‐solubility and self‐assembly capabilities, contradicting conventional assumptions. Notably, amphiphiles with chains of C18 or longer require only 0.3 wt.% for LLCs formation. These LLCs exhibit intriguing temperature‐dependent phase transitions, including a liquid‐like lamellar phase, a tilted gel phase, and a distinct phase characterized by tighter alkyl chain packing. The hydrocarbon chain length directly governs the transition temperatures and further influences the long‐range orientational ordering of lamellar sheets. Additionally, the tightly-packed configuration confers exceptional rheological properties, including ultra‐high viscosity, shear‐thinning behavior, and elasticity. These findings provide important insights for the design and engineering of high‐performance soft materials used in extreme environments.

DOI: 10.1002/anie.2025XXXXX

Table of Contents

1. Materials, characterizations and methods

2. Synthesis and characterization of alkyl alcoholamine amphiphiles

3. Appearance of alkyl diethanolamine amphiphiles dissolved in 1,2-propanediol/water

4. Structural parameters of lamellar liquid crystals obtained from SANS fitting results

5. Molecular dynamics simulation

6. Zero-shear viscosity as a function of C22-DEA concentration

1. Materials, characterizations and methods

***Materials.*** Lauryl diethanolamine (C12-DEA, >99%) was procured from Guangdong Wengjiang Chemical Reagent Co., LdD. (Shaoguan, China). Stearyl diethanolamine (C18-DEA, ≥ 98%) were supplied by Shanghai Adamas Reagent Co., Ltd. (Shanghai, China). 1-Bromotetradecane (>98%), 1-Bromohexadecane (>98%), 1-Bromoeicosane (>98%), 1-Bromodocosane (>98%), 2,2-Iminodiethanol (>99%), potassium iodide (>99%), anhydrous sodium carbonate (>99%), 1,2-propanediol (PG, ≥99 %), and deuterium oxide (99.9 atom% D) were all purchased from Shanghai Titan Scientific Co., Ltd. (Shanghai, China). The water used in this study was double-deionized via an ultrapure water purification system (CDUPT-III, Chengdu Ultrapure Technology Co., Ltd., China), achieving a resistivity of 18.25 MΩ·cm.

***Chemical structure characterization.*** Nuclear magnetic resonance (NMR) spectra were acquired at 30 °C using a Bruker Avance-Ⅱ 600 MHz NMR spectrometer. Chemical shifts (δ) are reported in parts per million (ppm), referenced to the internal standard protons of tetramethylsilane (TMS). Mass spectra were obtained on a Finnigan TSQ Quantum Ultra AM mass spectrometer. The purity was determined using a Waters 515 HPLC system equipped with an Alltech 2000 ELSD detector.

***Preparation of self-assembly system.*** A predetermined amount of alkyl diethanolamine was added to a 1,2-propanediol/water binary solvent and stirred at room temperature until the alkyl diethanolamine was completely dissolved. The resulting mixture was subsequently stored at room temperature for additional 24 h before further measurements.

***Determination of solubility.*** 0.5 wt% alkyl diethanolamine solutions were prepared in a 1,2-propanediol /glycerol cosolvent system. The transmittance of these solutions was measured using a UV-6100 double beam spectrophotometer (Shanghai Mapada Instruments Co. Ltd., China) at a fixed wavelength of 650 nm. The temperature of the solution was controlled through an external Julabo circulating bath with an accuracy of 0.1°C. Solubility was assessed based on the recorded transmittance values and visual observations.

***Small-angle neutron scattering measurement.*** Small-angle neutron scattering (SANS) measurements were conducted on the Small-Angle Neutron Diffractometer at China Spallation Neutron Source (CSNS, Dongguan, Guangdong Province). Incident neutrons with wavelengths ranging from 1 to 10 Å were selected by a double-disc bandwidth chopper, which was collimated to the specimen by a pair of apertures with a diameter of 6 mm. The sample-to-detector distance was 4 m, and the two-dimensional 3He tube array detector enabled coverage of *q* values ranging from 0.005 to 0.70 Å^−1^.

To optimize the contrast, the samples were prepared in a mixture of 1,2-propanediol /deuterium oxide. Measurements were conducted by loading the samples into clean disc-shaped quartz cells (Hellma) with a path length of 2 mm. For temperature-dependent measurements, the same sample was utilized continuously without substitution, with the temperature being reduced at a rate of 5 °C‧min^–1^. Before each test, the sample was allowed to stabilize for 10 min at the respective temperature.

The scattering data were normalized, corrected for transmission, and calibrated using a standard sample before being converted to absolute units. The SasView software (https://www.sasview.org/) was employed to fit the processed data. Lamellar structures were analyzed using the lamellar stack Caille model implemented in the software, which describes random lamellar head/tail/tail/head sheets with Caille structure factor. The scattering intensity *I*(*q*) is expressed as:

$I\left( q \right)=2\pi\frac{P\left( q \right)S(q)}{q^{2}\delta}$ (1)

where *P*(*q*) is the form factor, *S*(*q*) is the structure factor dependent on Caille constant, and δ is the total layer thickness. It should be noted that the assumptions of the model become invalid when the Caille parameter exceeds approximately 0.8 to 1.0.

***Small-angle X-ray scattering measurement.*** Small-angle X-ray scattering (SAXS) measurements were performed on a SAXSpace instrument (Anton Paar, Austria, Cu−Kα, λ = 0.154 nm), which was equipped with a Kratky block-collimation system and a Mythen detector. Prior to measurements, samples were loaded into a 1 mm diameter quartz capillary. Background scattering was accounted by recording the scattering profiles of the solvents within the same capillary under identical conditions. The data were normalized to the intensity of the incident primary beam, corrected for background scattering contributions, and analyzed using the SAXSquant software developed by Anton Paar.

***Wide-angle X-ray scattering measurements.*** The wide-angle X-ray scattering (WAXS) measurements were conducted using a synchronous diffraction laboratory instrument, the Nano-inXider (Xenocs, France). This instrument is equipped with a micro-focus source that generates X-rays with a wavelength of λ = 1.542 Å (Genix3D), operating at 50 kV and 0.6 mA. Each sample was exposed to the X-ray beam for 600 s using the Nano-inXider and 1 s at the synchrotron. The solution samples were transferred into borosilicate capillaries (1.5 mm outer diameter, 0.01 mm wall thickness) with a sample-to-detector distance of 79 mm. For temperature-dependent measurements, the sample was used without replacement, and the temperature was decreased at a rate of 5 °C‧min^–1^. Prior to testing, the sample was equilibrated for 10 min at each temperature.

***Cryogenic scanning electron microscopy observation.*** Cryogenic scanning electron microscopy (cryo-SEM) analyses were carried out using a FEI Quanta 450 scanning microscope (FEI Company, USA), which was fitted with a Quorum cryo-stage PP3000T. Specimens for examination were processed via freeze-drying: initially, they were frozen in liquid nitrogen at −185 °C for 30 s, followed by transfer to a chamber where moisture was sublimated under vacuum conditions at −90 °C for 10 min. Afterwards, the specimens were sputter-coated with gold and examined at −140 °C with an accelerating voltage of 5 kV.

***Polarizing optical microscopy visualization.*** The crystalline morphologies were analyzed using a Nikon LV100NPOL polarizing optical microscope (Nikon Corporation, Japan) under room temperature conditions. Samples were observed with polarized light rotated by two degrees. Each sample was placed in a slide groove designed for high transmittance to maintain stable solution surfaces and phase states. The polarizing microscope images were captured at a magnification of 10x or 20x under consistent lighting conditions.

***Rheological test.*** Rheological measurements were performed using a Physica MCR 302 rotational rheometer (Anton Paar, Austria) equipped with CC27 concentric cylinder geometry. Prior to testing, all samples were allowed to equilibrate at the specified temperature for 10 min. Steady shear viscosity data were recorded over a shear rate range of 10^−3^ to 10^3^ s^−1^, with the rate increasing logarithmically. Oscillatory shear tests were conducted within the linear viscoelastic region, which had been previously identified through dynamic stress sweep analyses. All experiments were executed in stress-control mode, and the instrument was calibrated with standard oil prior to the measurements.

***Statistical Analysis.***

SAXS data are normalized using SAXSquant. The image normalization of 2D WAXS pattern was performed using FIT2D. The results of molecular dynamics simulations are obtained from 20 ns trajectory statistics. The hydrogen bonds are presented as mean ± standard deviation (SD).

2. Synthesis and characterization of alkyl alcoholamine amphiphiles

C14-DEA, C16-DEA, C20-DEA, and C22-DEA were synthesized following an identical one-pot procedure. The synthesis involved the reaction of corresponding brominated alkanes and 2,2-iminodiethanol in the presence of anhydrous potassium carbonate and potassium iodide in at 80 °C, as depicted in Figure S1a. The synthetic process is illustrated using C14-DEA as an example. In a three-necked reaction flask, 1-bromotetradecane, diethanolamine, anhydrous potassium carbonate, and potassium iodide were added at a molar ratio of 1:1.5:2:0.2. Nitrogen gas was introduced to purge the air from the reaction vessel, followed by the addition of acetonitrile as the solvent. The reaction mixture was heated to 80 °C and maintained under reflux conditions for 12 h. Upon completion of the reaction, the solvent acetonitrile was removed through rotary evaporation to yield the crude product. Subsequently, dichloromethane and water at the volume ratio of 1:1 was respectively added to the crude product. The resulting biphasic mixture was separated, allowing the removal of excess diethanolamine in the aqueous phase. The dichloromethane solution was collected, dried over anhydrous sodium sulfate, and concentrated by rotary evaporation to afford pure C14-DEA.

Figures S1b-c present their ^1^H NMR and ^13^C NMR spectra, respectively. It is evident that the spectra exhibit high similarity due to their analogous chemical structures. In the ^1^H NMR spectra, the peak at 3.60 ppm corresponds to the protons on the methylene group adjacent to the hydroxyl group. The peaks at 2.70 ppm and 2.55 ppm are assigned to the protons on the methylene group bonded to the nitrogen (*N*) atom, while the peak at 1.45 ppm is attributed to the protons on the secondary methylene group near the *N* atom in the hydrocarbon tail. The peak at 0.85 ppm corresponds to the terminal methyl group, whereas the intense peak at 1.25 ppm represents the protons on the methylene groups within the hydrocarbon tail. Moreover, the peaks in the ^13^C NMR spectra are well-corelated with the carbon atoms of the synthesized amphiphiles, and the molecular weight determined by mass spectrometry aligns with the theoretical molecular weight (Figure S2), thereby confirming the successful synthesis of the four amphiphiles. In addition, HPLC analysis (Figure S3) reveals that the purity of all these four surfactants is between 95.0% and 99.2%.

C14-DEA: Yellow waxy with yield of 83.2 % and purity of 99.1% (determined by HPLC, Figure S3a). ^1^H NMR (600 MHz, CDCl_3_, δ, ppm): 3.96 (s, 2H, OH), 3.63−3.61 (t, 4H, −CH_2_−OH), 2.72−2.70 (t, 4H, N−(CH_2_)_2_), 2.59−2.56 (t, 2H, CH_2_−N), 1.46 (s, 2H, CH_2_−CH_2_−N), 1.25−1.21 (m, 24H, −CH_2_−), 0.85−0.82 (t, 3H, CH_3_). ^13^C NMR (151 MHz, CDCl_3_, δ, ppm): 59.80, 56.21, 54.95, 32.06, 29.83−29.78, 29.71, 29.50, 27.53, 27.22, 22.83, 14.26. ESI-HRMS (Figure S2a): Calcd.: 302.31 (M^+^); Found: m/z =302.36.

C16-DEA: White waxy with yield of 97.5 % and purity of 99.2% (Figure S3b). ^1^H NMR (600 MHz, CDCl_3_, δ, ppm): 3.65−3.63 (t, 4H, −CH_2_−OH), 2.70−2.68 (t, 4H, N−(CH_2_)_2_), 2.56−2.54 (t, 2H, CH_2_−N), 1.48 (s, 2H, CH_2_−CH_2_−N), 1.30−1.25 (m, 28H, −CH_2_−), 0.89−0.87 (t, 3H, CH_3_). ^13^C NMR (151 MHz, CDCl_3_, δ, ppm): 59.80, 56.20, 54.95, 29.84−29.78, 29.72, 29.50, 27.53, 27.23, 22.83, 14.26. ESI-HRMS (Figure S2b): Calcd.: 330.34 (M^+^); Found: m/z =330.33.

C20-DEA: Yellow waxy with yield of 85.2% and purity of 98.8% (Figure S3c). ^1^H NMR (600 MHz, CDCl_3_, δ, ppm): 3.61−3.59 (t, 4H, −CH_2_−OH), 2.65−2.63 (t, 4H, N−(CH_2_)_2_), 2.52−2.49 (t, 2H, CH_2_−N), 1.44 (s, 2H, CH_2_−CH_2_−N), 1.26−1.24 (m, 36H, −CH_2_−), 0.87−0.85 (t, 3H, CH_3_). ^13^C NMR (151 MHz, CDCl_3_, δ, ppm): 59.82, 56.21, 54.95, 32.07, 29.85−29.79, 27.53, 27.25, 22.83, 14.26. ESI-HRMS (Figure S2c): Calcd.: 386.40 (M^+^); Found: m/z =386.33.

C22-DEA: Yellow waxy with yield of 89.5% and purity of 95.0% (Figure S3d). ^1^H NMR (600 MHz, CDCl_3_, δ, ppm): 3.63−3.61 (t, 4H, −CH_2_−OH), 2.69−2.67 (t, 4H, N−(CH_2_)_2_), 2.56−2.53 (t, 2H, CH_2_−N), 1.46 (s, 2H, CH_2_−CH_2_−N), 1.28−1.22 (m, 40H, −CH_2_−), 0.86−0.84 (t, 3H, CH_3_). ^13^C NMR (151 MHz, CDCl_3_, δ, ppm): 59.77, 56.21, 54.95, 32.07, 29.84−29.72, 27.53, 27.19, 22.83, 14.26. ESI-HRMS (Figure S2d): Calcd.: 414.43 (M^+^); Found: m/z =414.39.


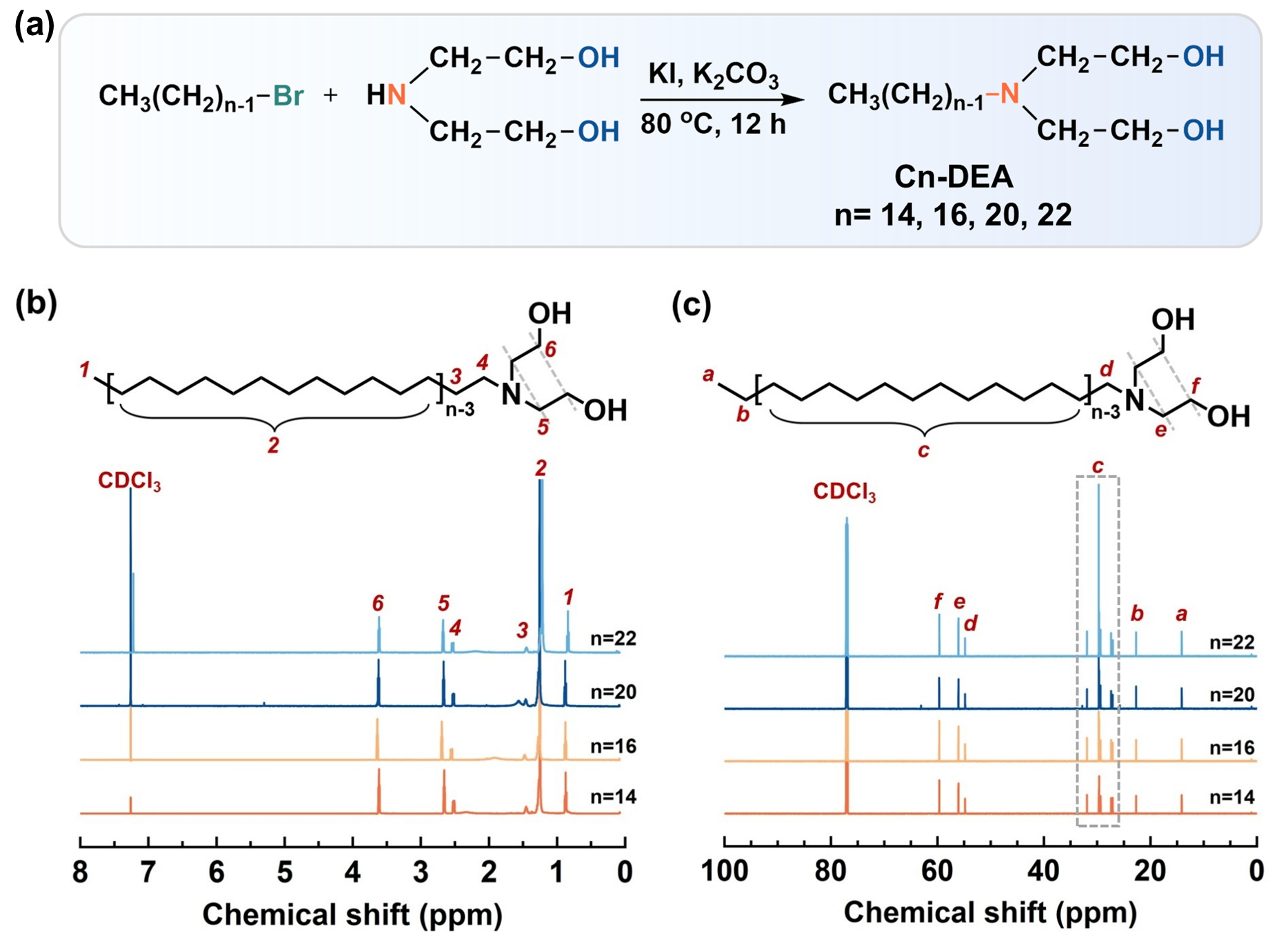


**Figure S1.** (a) Synthetic route of alkyl alcoholamine amphiphiles. (b) ^1^H NMR and (c) ^13^C NMR spectra of C14-DEA, C16-DEA, C20-DEA, and C22-DEA in CDCl_3_.

**(a)**

**(b)**

**(c)**

**(d)**

**Figure S2.** Mass spectra of (a) C14-DEA, (b) C16-DEA, (c) C20-DEA, and (d) C22-DEA.


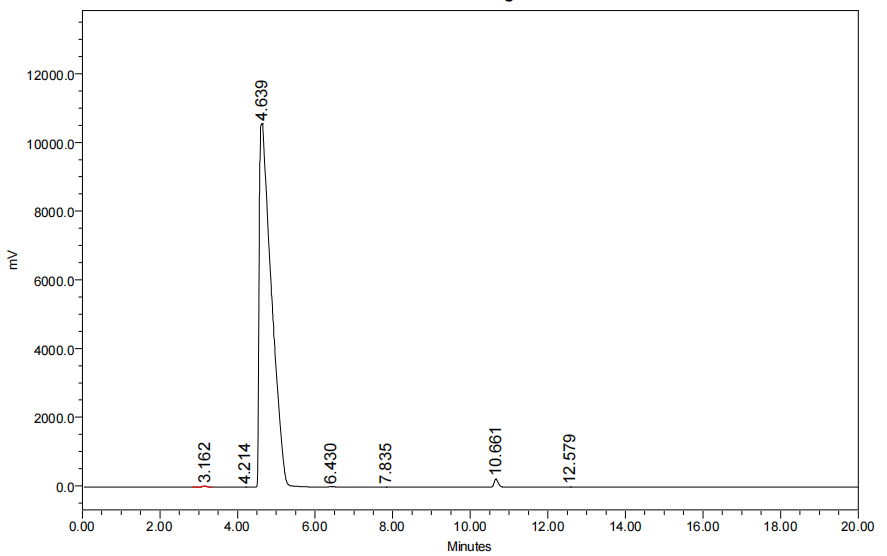

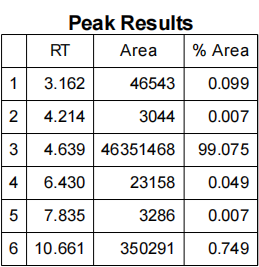

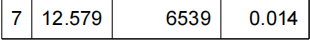


**(a)**


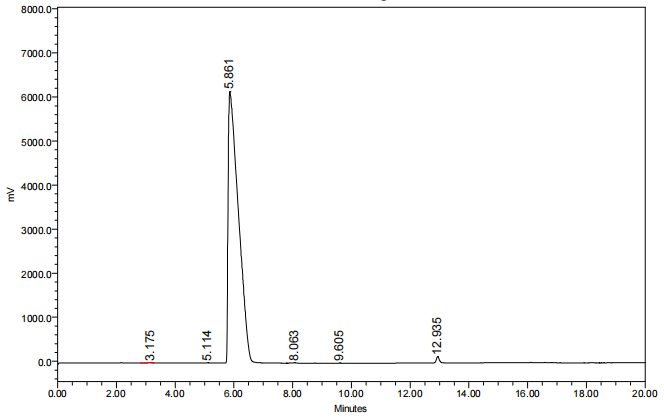


**(b)**


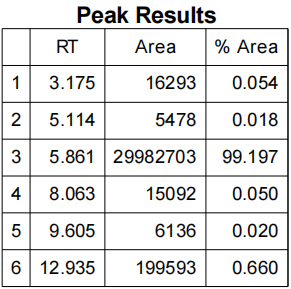

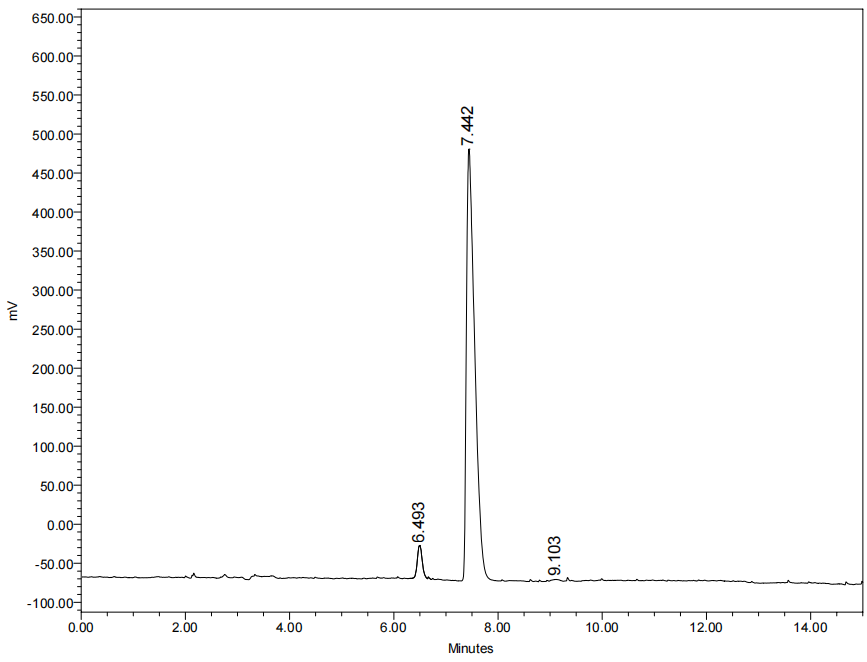

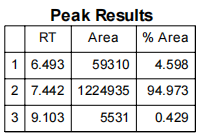


**(d)**


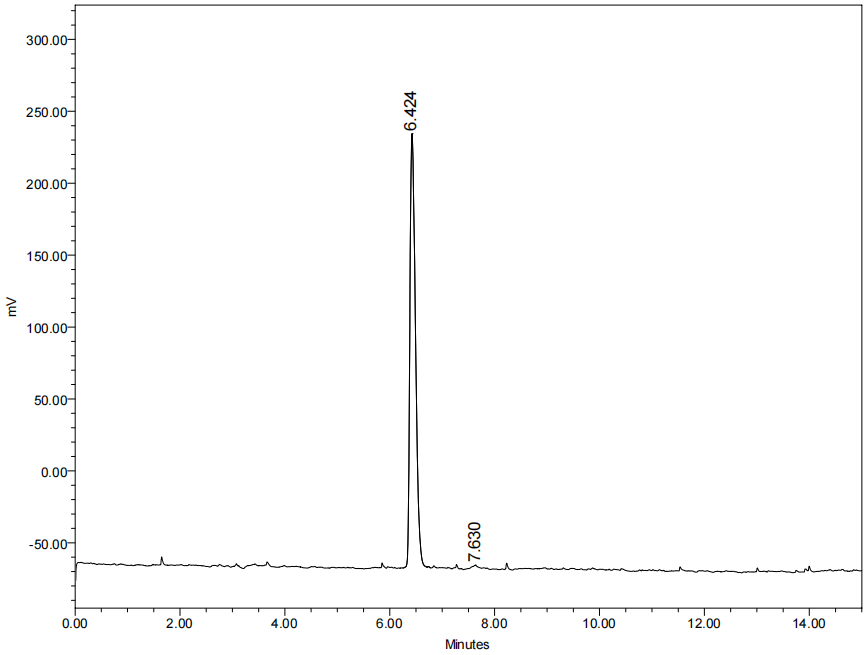

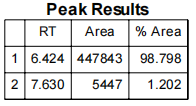


**(c)**

**Figure S3.** HPLC spectra of (a) C14-DEA, (b) C16-DEA, (c) C20-DEA, and (d) C22-DEA.

3. Appearance of alkyl diethanolamine amphiphiles dissolved in 1,2-propanediol/water


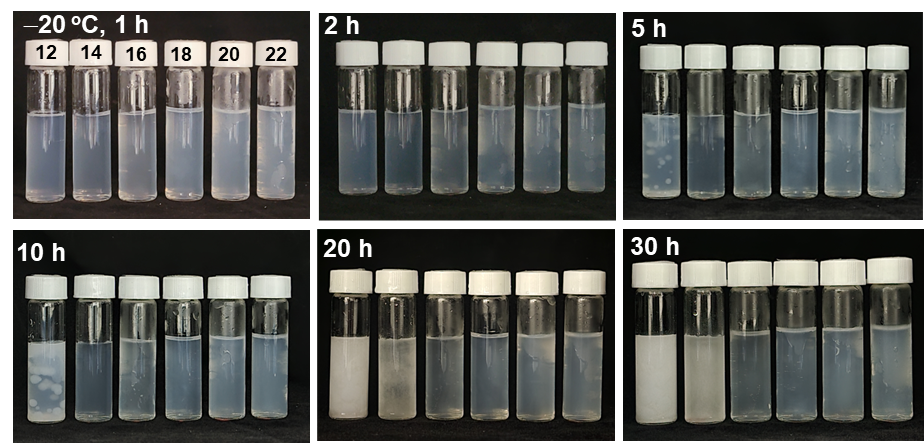


**Figure S4.** Appearance of 0.5 wt% alkyl diethanolamine amphiphiles (Cn-DEA) in 1,2-propanediol/water (50/50, vol%/vol%) at −20 °C last for 1 h, 2 h, 5 h, 10 h, 20 h, and 30 h, respectively (n=12, 14, 16, 18, 20, 22).

4. Structural Parameters of lamellar liquid crystals obtained from SANS fitting results

**Table S1**. Structural Parameters for the lamellar stack Caille model used for fitting SANS data acquired for 5.0 wt% alkyl diethanolamine amphiphiles in 1,2-propanediol/deuterium oxide (50/50, vol%) at 20 °C.

| Amphiphile | *d*_c_ (nm) | *d*_so_ (nm) | *d*_sp_ (nm) | Caille parameter |
| --- | --- | --- | --- | --- |
| C16-DEA | 1.5 | 40.2 | 41.7 | 0.24 |
| C18-DEA | 2.3 | 52.9 | 55.2 | 0.07 |
| C20-DEA | 3.1 | 54.7 | 57.8 | 0.14 |
| C22-DEA | 3.1 | 58.9 | 62.0 | 0.12 |

*d*_c_ denotes hydrophobic bilayer thickness, *d*_so_ refers to the solvent layer thickness between two bilayers, *d*_sp_ means interlamellar spacing.

**Table S2**. Structural Parameters for the lamellar stack Caille model used for fitting SANS data acquired for 5.0 wt% C22-DEA in 1,2-propanediol/deuterium oxide (50/50, vol%) at various temperatures.

| *T* (°C) | *d*_c_ (nm) | *d*_so_ (nm) | *d*_sp_ (nm) | Caille parameter |
| --- | --- | --- | --- | --- |
| 80 | 3.5 | 64.7 | 68.2 | 0.15 |
| 60 | 3.4 | 61.9 | 65.3 | 0.12 |
| 20 | 3.1 | 58.9 | 62.0 | 0.12 |
| –20 | 3.1 | 55.2 | 58.3 | 0.8 |

*d*_c_ denotes hydrophobic bilayer thickness, *d*_so_ refers to the solvent layer thickness between two bilayers, *d*_sp_ means interlamellar spacing.

5. Molecular dynamics simulation

Figure S5 shows the MD simulation system for C12-DEA, C18-DEA, and C22-DEA bilayer formation in 1,2-propanediol/water binary solvent. The system contains 200 DEA molecules (100 per side) forming a bilayer. The hydrophilic groups face outward, and the hydrophobic chains face inward. The solvent is 1,2-propanediol/water mixture at the volume ratio of 50:50. As shown in Figure S6, the bilayer system construction followed a four-step protocol: (1) Cn-DEA molecules were arranged in a standard bilayer configuration. (2) 1,2-propanediol/water mixtures were inserted symmetrically on both bilayer interfaces; (3) System energy minimization was performed to optimize the initial structure. (4) Bilayer simulations were performed under NPT conditions and the relevant data were statistically analyzed. The simulation box size is 6.56 × 6.56 × 13.70 nm³. Periodic boundary conditions are applied in all directions. We used the OPLS all-atom force field^[1]^ for alkyl diethanolamine amphiphiles and 1,2-propanediol. The structural files and force field parameters for alkyl diethanolamine amphiphiles and 1,2-propanediol are uploaded in the attached compressed file. The TIP3P model^[2]^ was used for water. The Lorentz-Berthelot mixing rule calculated interactions between atoms.^[3]^ All simulations were performed using Gromacs software.^[4]^ A Nose-Hoover thermostat controlled the temperature (−20°C, 0°C, and 20°C). The Leap-Frog algorithm integrated Newton’s equations with a 2 fs timestep, while electrostatic interactions were resolved using a 0.1 nm FFT grid. After a 50 ns NPT equilibrium simulation at 1 atm to form the bilayer, a 20 ns production run was performed for data analysis.


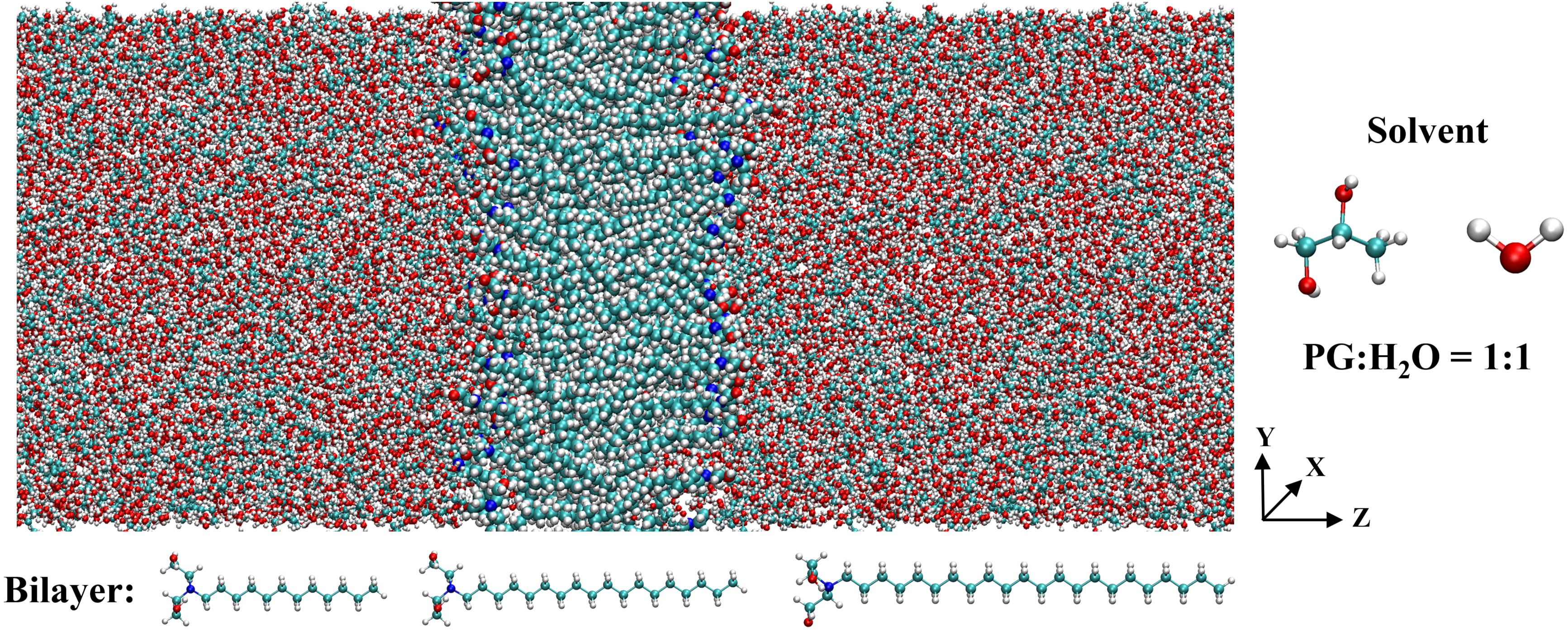


**Figure S5.** The schematic diagram of molecular dynamics simulations for C12-DEA, C18-DEA, and C22-DEA in 1,2-propanediol/water (50/50, vol%) binary solvent.


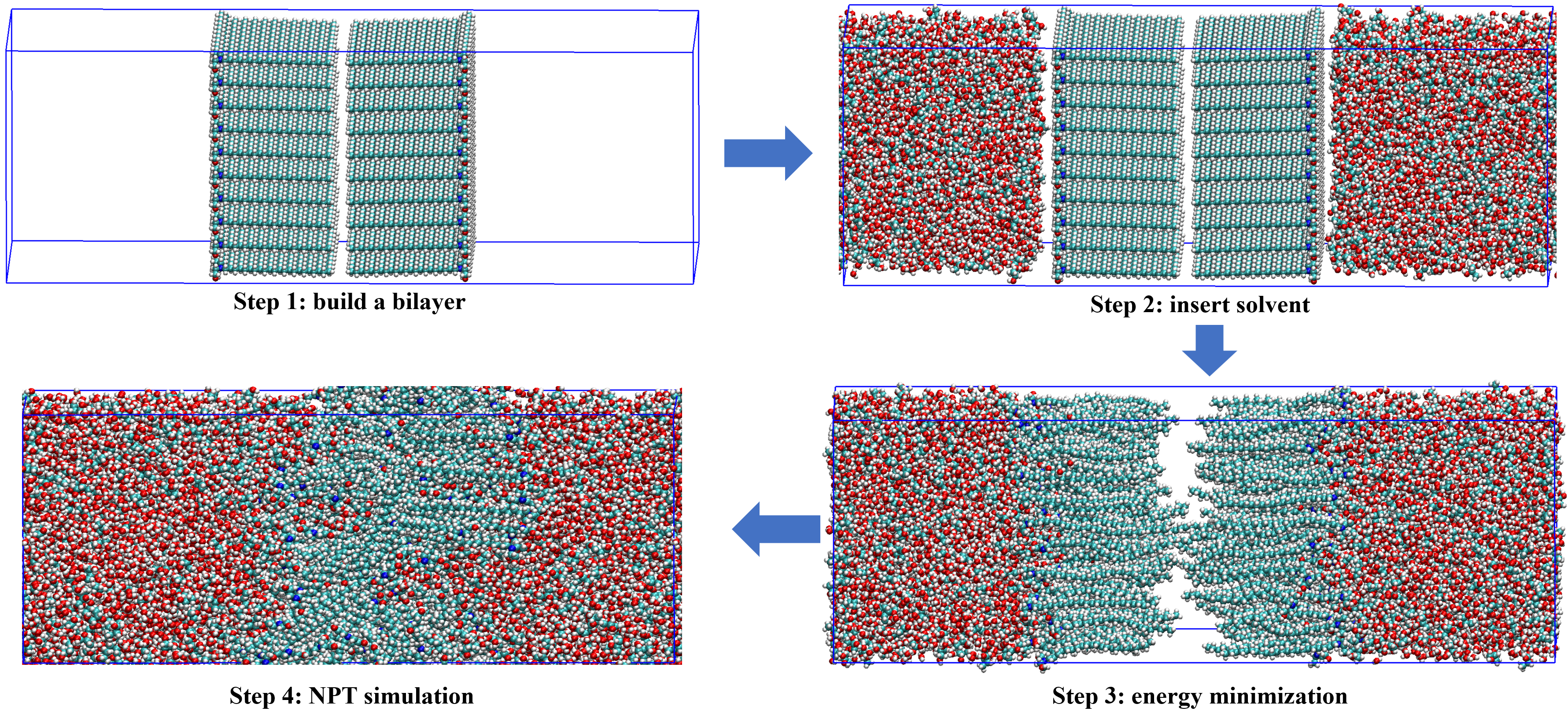


**Figure S6.** Schematic diagram of the modeling process for a bilayer system.


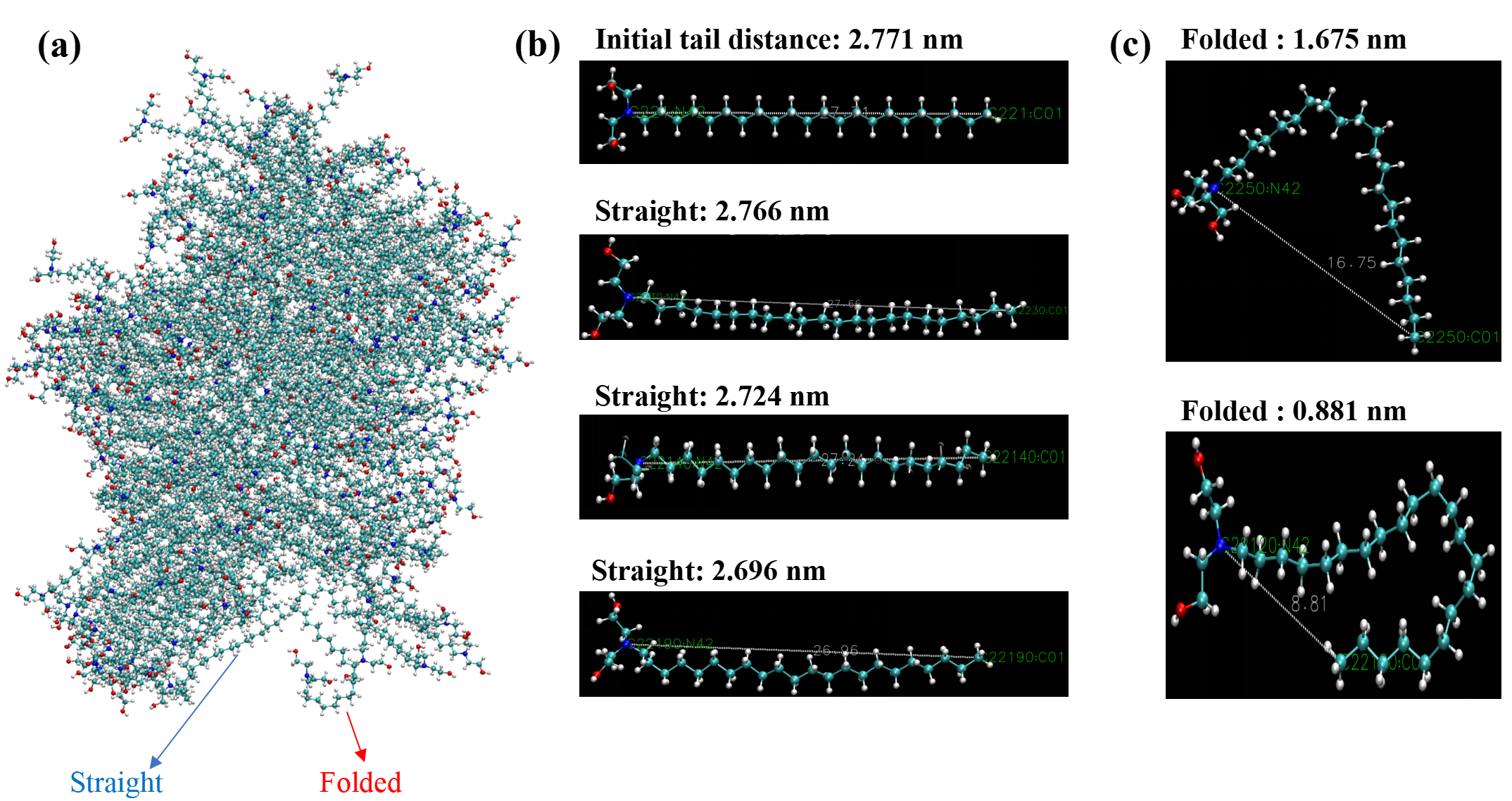


**Figure S7.** The hydrocarbon tail configuration of C22-DEA. (a) C22-DEA in a bilayer. (b) Straight C22-DEA. (c) Folded C22-DEA. The image is displayed using VMD software, and the distance units on the image are angstroms.

Figure S7 illustrates the configuration of the C22-DEA tail chain. It is evident that the bilayer structure deviates from the ideal bilayer arrangement observed in the initial configuration. The tail chains of C22-DEA exhibit a high degree of flexibility, enabling them to intertwine within the bilayer. To provide a clearer depiction of the various tail chain configurations, individual molecular structure of C22-DEA was extracted, incuding the initial configuration, the extented (straight) tail chain (Figure S7b), and the folded tail chain (Figure S7c).


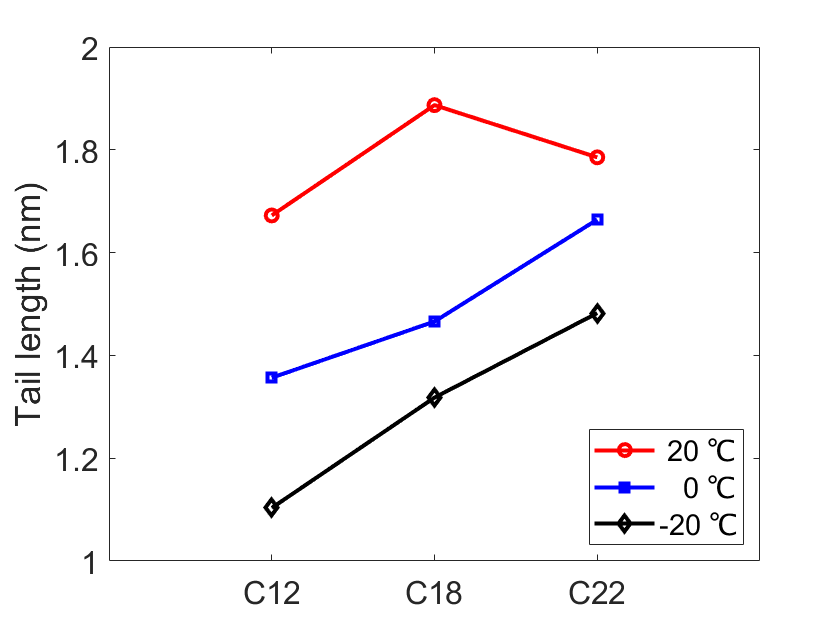


**Figure S8.** Hydrocarbon tail length of C12-DEA, C18-DEA, and C22-DEA at different temperatures obtained from molecular dynamic simulation.

6. Zero-shear viscosity as a function of C22-DEA concentration


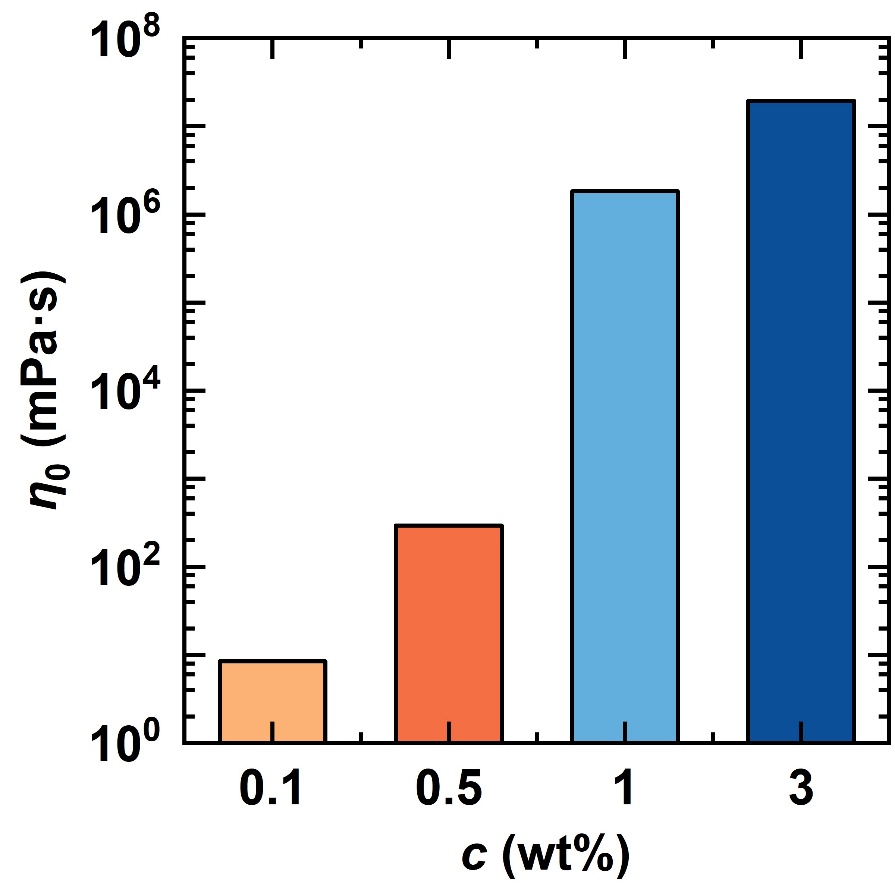


**Figure S9.** Zero-shear viscosity as a function of C22-DEA concentration in 1,2-propanediol/water (50/50, vol%) at 20 °C.

# References

[1] W. L. Jorgensen, J. Tirado-Rives, *Proc. Natl. Acad. Sci.* **2005**, *102*, 6665−6670.

[2] P. Mark, L. Nilsson, *J. Phys. Chem. A* **2001**, *105*, 9954−9960.

[3] H. A. Lorentz, *Ann. Phys. Berlin*, **1881**, *248*, 127−136.

[4] B. Hess, C. Kutzner, D. van der Spoel, E. Lindahl, *J. Chem. Theory Comput.* **2008**, *4*, 435−447.

# Author Contributions

Weiluo Guo: Investigation, data curation, validation

Zhenghua Sun: Investigation, data curation, formal analysis, writing of original draft

Runxi Wang: Investigation, methodology, validation, Writing – review & editing

Zhuo Zheng: Methodology, investigation

Yubin Ke: Methodology, resources

Hua Yang: Investigation, methodology

Lingzhi Xie: Methodology, resources

Yujun Feng: Funding acquisition, resources

Hongyao Yin: Conceptualization, validation, funding acquisition, project administration, writing of original draft, Writing – review & editing
